# Multi-Element Composition of Wild *Prunus spinosa* Fruits Across Contrasting Environments: Implications for Food Safety and Quality

**DOI:** 10.3390/foods15101726

**Published:** 2026-05-14

**Authors:** Andra Ioana Vlad, Szilárd Bartha, Voichița Timiș-Gânsac, Laviniu Ioan Nuțu Burescu, Tunduc Adrian, Mariana Florica Bei, Florin Alexandru Rebrean, Călugăr Anamaria, Petrică Tudor Moțiu, Florin-Dumitru Bora

**Affiliations:** 1 Department of Food Engineering, University of Oradea, 1 University Street, 410087 Oradea, Romania; iavlad@uoradea.ro (A.I.V.); mbei@uoradea.ro (M.F.B.); 2 Department of Forestry and Forest Engineering, University of Oradea, 1 University Street, 410087 Oradea, Romania; sbartha@uoradea.ro (S.B.); vtimis@uoradea.ro (V.T.-G.); lburescu@uoradea.ro (L.I.N.B.); atunduc@uoradea.ro (T.A.); 3 Department of Forestry, Faculty of Forestry and Cadastre, University of Agricultural Sciences and Veterinary Medicine, 3–5 Mănăştur St., 400372 Cluj-Napoca, Romania; florin-alexandru.rebrean@usamvcluj.ro; 4 Viticulture and Oenology Department, Advanced Horticultural Research Institute of Transylvania, Faculty of Horticulture and Business in Rural Development, University of Agricultural Sciences and Veterinary Medicine Cluj-Napoca, 3–5 Mănăştur Street, 400372 Cluj-Napoca, Romania; anamaria.calugar@usamvcluj.ro; 5 Laboratory of Chromatography, Advanced Horticultural Research Institute of Transylvania, Faculty of Horticulture and Business for Rural Development, University of Agricultural Sciences and Veterinary Medicine, 400372 Cluj-Napoca, Romania

**Keywords:** *Prunus spinosa*, environmental contamination, heavy metals, pollution gradient, ICP-MS, bioindicator, anthropogenic pressure, trace elements, food safety

## Abstract

Environmental contamination with potentially toxic elements is a growing concern for ecosystem quality and food safety. This study evaluated the relationships between environmental conditions, anthropogenic activities, and the elemental composition of *Prunus spinosa* fruits collected from western and central Romania along a pollution gradient. Eighty samples from ten sites representing non-polluted, agricultural, traffic-exposed, and mining-affected areas were analyzed by ICP-MS after microwave digestion. Fruits from impacted areas showed compositional differences, including lower concentrations of some essential macroelements and higher levels of several trace elements potentially associated with anthropogenic pressure. Increased sodium, aluminum, and silicon contents were consistent with environmental stress and enhanced environmental exposure and possible soil-derived particulate influence, while boron and molybdenum declined with pollution intensity. Elemental patterns were mainly associated with local environmental conditions and appeared consistent with site-specific environmental influences. Food safety assessment indicated generally low to moderate risk depending on sampling origin. Overall, *Prunus spinosa* fruits showed potential as a bioindicator of environmental quality and a useful tool for monitoring anthropogenic contamination.

## 1. Introduction

Environmental contamination by potentially toxic elements is a major environmental and public health concern because of its adverse effects on ecosystems and human populations [1]. These elements derive from both natural geochemical sources and anthropogenic activities, including agriculture, industrial emissions, traffic, and mining [2]. Once released, they may persist in soils and be taken up by plants, providing a pathway into the food chain [3,4,5,6]. Consequently, the elemental composition of plant-derived foods, including wild fruits, can reflect local environmental conditions and contamination levels [7,8,9,10]. Therefore, assessing elemental accumulation in edible plant species is relevant for evaluating environmental quality and potential risks to consumers [11,12].

*Prunus spinosa* L. is a widely distributed spontaneous species across Europe, including Romania, with high ecological plasticity and adaptation to diverse environmental conditions [13,14,15,16]. It occurs in habitats ranging from forest ecosystems to agricultural and anthropogenically influenced areas, including zones affected by traffic and mining [16,17,18,19]. Its edible fruits have nutritional and economic relevance [20,21,22,23]. Owing to its ability to accumulate chemical elements in response to environmental conditions, *P. spinosa* is considered a valuable bioindicator species [24,25,26,27,28]. Its occurrence across contrasting habitats makes it particularly suitable for assessing spatial variability in elemental composition and environmental contamination [29,30,31,32].

Numerous studies have shown that wild plants and fruits can accumulate metals and other chemical elements in relation to environmental conditions and contamination levels [33,34,35,36,37]. Increasing attention has been given to the simultaneous determination of macroelements, essential microelements, and potentially toxic elements because of their relevance for nutrition, ecology, and food safety [38,39,40]. Plant elemental composition is influenced by geological substrate, soil properties, land use, agricultural practices, and pollution sources such as traffic and mining [41,42,43,44,45]. However, studies on *Prunus spinosa* remain limited, often focusing on a restricted number of elements or specific ecological contexts, with few systematic evaluations across well-defined environmental gradients [46,47,48,49,50,51,52,53,54]. In addition, integrated approaches linking biomonitoring, geochemical interpretation, and food safety assessment remain scarce [6]. Elemental accumulation in edible fruits is also relevant because these matrices may represent a pathway of human exposure, particularly for toxic elements such as Pb and Cd [1,2,4,6,35,36]. Since regulatory limits are unavailable for many elements, concentrations should be interpreted in relation to contamination sources, element mobility, and environmental context [3,11,41,42,55]. Therefore, comprehensive comparative studies linking environmental pressure with multi-elemental profiles are still needed [54,55,56]. To our knowledge, this study provides the first integrated ICP–MS multi-element assessment of wild *Prunus spinosa* fruits across contrasting anthropogenic environments in Romania, including natural, agricultural, traffic-influenced, and mining-affected areas.

We investigated the influence of environmental conditions and anthropogenic pressure on the multi-elemental composition of *Prunus spinosa* fruits collected from western and central Romania. Sampling sites were selected along a well-defined pollution gradient, including minimally impacted natural areas, agricultural landscapes, traffic-influenced zones, and regions affected by historical mining activities. This stratified design enabled the assessment of environmental heterogeneity and its relationship with elemental accumulation. Specifically, the objectives were: (i) to quantify macroelements, essential microelements, and trace and potentially toxic elements using ICP–MS; (ii) to evaluate spatial variation across contrasting sites; and (iii) to assess the potential of *P. spinosa* fruits as bioindicators of environmental contamination. We hypothesized that elemental concentration patterns would reflect site-specific environmental conditions and anthropogenic influences, resulting in distinct compositional signatures along the pollution gradient.

## 2. Materials and Methods

### 2.1. Study Area and Sampling Sites

The study was conducted in western and central Romania (Alba, Bihor, and Cluj counties), covering an environmental gradient from lowland agricultural areas (~150 m a.s.l.) to mountainous regions in the Apuseni Mountains (~900 m a.s.l.). Geographic coordinates were recorded using the WGS84 system, and altitude values were derived from digital elevation models. Ten sampling sites were selected (Table S1), representing diverse habitat types and levels of anthropogenic influence, including non-polluted mountain areas (e.g., Gârda de Sus, Ponor), forest ecosystems, agricultural zones (Leș), traffic-affected sites (Bucea), and a mining-impacted area (Zlatna).

Plant material consisted of fully mature fruits of *Prunus spinosa* L., collected during the peak ripening period from spontaneous populations under comparable ecological conditions (field margins, forest edges, grasslands, and roadside areas), with a standardized phenological stage.

Sampling sites were preliminarily classified into four categories (non-polluted, low impacted, moderately impacted, and polluted) using a multi-criteria framework based on land use characteristics, proximity to potential contamination sources, including locations within 500 m of major roads, settlements, or industrial areas, agricultural intensity, traffic exposure, and documented historical mining activity where applicable (Table S2). Supporting spatial information was obtained from field observations, topographic maps, and regional land-cover datasets. This stratified design enabled the evaluation of environmental drivers of macro-, micro-, and trace element accumulation in *Prunus spinosa* fruits.

### 2.2. Plant Material and Sampling Procedure

The plant material consisted of fully mature fruits of *Prunus spinosa* L., collected during the peak fruiting period (October–November 2025) to ensure physiological uniformity and comparability. At each sampling site, fruits were collected using a randomized approach from spontaneously growing shrubs. Each biological replicate consisted of pooled fruits from a different individual shrub, ensuring biological independence and avoiding pseudoreplication.

The sampling effort was defined to provide adequate statistical power for detecting differences in elemental composition among sampling sites and pollution categories. The required sample size was estimated based on power analysis for a one-way ANOVA design:

*N* = *f*(*α*, 1 − *β*, *f*)

where *α* = 0.05 (significance level), 1 − *β* = 0.80 (statistical power), and effect size was expressed as Cohen’s *f*. Assuming a medium effect size (*f* = 0.25), the minimum required sample size for a balanced design was estimated at approximately 45–52 biological samples. Given the inclusion of 10 sampling sites, this minimum requirement corresponds to approximately 5 replicates per site:

50 samples/10 sites = 5 replicates per site

To improve statistical robustness and better capture within-site variability, the replication level was increased beyond the minimum requirement. A total of 80 biological samples was therefore collected, corresponding to:

80 samples/10 sites = 8 replicates per site

Thus, the final sampling design consisted of

10 sites × 8 replicates = 80 biological samples

This level of replication improves variance estimation, increases statistical power, and supports both univariate (ANOVA) and multivariate analyses. A total of 80 biological samples (8 replicates per site) were collected from 10 sampling sites across Alba, Bihor, and Cluj counties. Sites were classified into four categories of environmental pressure (non-polluted, low impacted, moderately impacted, and polluted) based on land use characteristics, proximity to potential contamination sources, and overall anthropogenic pressure. The sampling design is summarized in Table 1, while detailed site characteristics are provided in Table S3.

### 2.3. Sample Preparation

Collected *Prunus spinosa* L. fruit samples were subjected to a consistent sample preparation procedure including washing, drying, grinding, and storage (Table S4). Fresh samples were washed with distilled water (repeated rinsing and gentle agitation) to remove surface contaminants and air-dried with filter paper at room temperature.

Samples were oven-dried at 40–60 °C for 24–72 h until constant weight was achieved, with moisture removal verified by periodic weighing using an analytical balance (Radwag AS 220.R2, Radwag, Radom, Poland). Dried samples were cooled in a desiccator (Duran Group, Mainz, Germany), ground to a fine powder using a laboratory mill (Retsch Grindomix GM 200, Retsch GmbH, Haan, Germany), and, when necessary, sieved (~0.5 mm) to ensure homogeneity. All equipment was cleaned between samples using distilled water and ethanol to prevent cross-contamination. Prepared samples were stored in airtight polyethylene containers (Nalgene, Rochester, NY, USA), protected from light and humidity, at room temperature or 4 °C until analysis.

Prepared samples were stored in airtight polyethylene containers (Nalgene, Rochester, NY, USA), protected from light and humidity, at room temperature or 4 °C until analysis. The sample preparation procedure was applied uniformly to all collected samples to ensure analytical consistency and minimize cross-contamination risks.

### 2.4. Reagents and Chemicals

All reagents were of high purity and used without further purification. Nitric acid (HNO_3_, 65%) and hydrogen peroxide (H_2_O_2_, 30%) were obtained from Merck KGaA (Darmstadt, Germany). Ultrapure water (resistivity ≥ 18.2 MΩ·cm) was produced using a Milli-Q^®^ system (Merck Millipore, Darmstadt, Germany). Multi-element calibration standards (Certipur^®^) and Hg single-element standards (TraceCERT^®^) were supplied by Sigma-Aldrich (St. Louis, MO, USA). Analytical accuracy was verified using a certified reference material (NIST SRM 1573a, Tomato Leaves; National Institute of Standards and Technology, Gaithersburg, MD, USA). All glassware (DURAN Group, Mainz, Germany) and plasticware (Nalgene, Rochester, NY, USA) were acid-washed (10% HNO_3_) and rinsed with ultrapure water prior to use.

### 2.5. Digestion Procedure

Sample digestion was performed using a closed-vessel microwave-assisted system (START D, Milestone Srl, Sorisole, Italy) equipped with PTFE vessels. Approximately 0.300–0.500 g of dried homogenized sample was digested with HNO_3_ (65%) and H_2_O_2_ (30%) after a short pre-digestion step at room temperature. Digests were diluted with ultrapure water after cooling. All samples were analyzed in triplicate, consisting of three independent microwave digestions performed from the same homogenized biological sample, followed by separate ICP-MS measurements for each digest. Reagent blanks were included in each analytical batch. Detailed digestion conditions are provided in Table S5 (Supplementary Material).

### 2.6. Elemental Analysis

Elemental analysis was performed by inductively coupled plasma mass spectrometry (ICP-MS) following microwave-assisted digestion. Samples were analyzed using an ICP-MS system (Thermo Scientific iCAP Q, Bremen, Germany). Quantification was achieved by external calibration with multi-element standards in a nitric acid matrix, while Hg was determined using a single-element standard. Measurements were performed in helium collision mode (KED) to reduce polyatomic interferences, with optimized isotope selection. Particular attention was given to elements susceptible to spectral interferences and memory effects, especially Sb, Sn, Cr, and Ni. Measurements were performed in He-KED collision mode using a helium gas flow of approximately 4.5 mL/min. Instrument performance was monitored daily through oxide and doubly charged ion formation, with CeO^+^/Ce^+^ maintained below 2% and Ce^2+^/Ce^+^ below 3%. Internal standards (^103^Rh, ^115^In, and ^209^Bi) showed signal deviations within ±3.2–4.8% during analytical sequences, confirming stable correction of instrumental drift and matrix-related signal suppression. Calibration verification standards analyzed every 10–15 samples showed deviations below 5%. Carry-over and memory effects were minimized using 2–5% HNO_3_ rinse solutions between samples, while procedural blanks and periodic calibration verification were used to monitor potential residual signals during analytical sequences.

Carry-over and memory effects were minimized using 2–5% HNO_3_ rinse solutions between samples. Additional carry-over assessment was performed through rinse blank measurements and calibration verification standards analyzed immediately after high-concentration samples and multi-element calibration standards. Residual analyte signals measured in post-rinse blanks remained below 1.8–4.6% of the corresponding LOQ values and below the respective background-equivalent concentration (BEC) levels for all investigated elements, including memory-prone analytes such as Pb, Cd, Sb, Sn, Cr, and Ni. Calibration verification recoveries following high-concentration samples remained within ±3.7–4.9% of expected values, while internal standard recoveries (Rh, In, and Bi) remained stable within ±3.2–4.8% throughout the analytical sequence. No progressive signal accumulation, residual peak persistence, or systematic blank elevation was observed during consecutive ICP-MS measurements, indicating negligible carry-over effects and absence of analytically significant cross-sample contamination.

All analyses were carried out in triplicate, including reagent blanks for quality control. Elemental concentrations were expressed as mg/kg dry weight (DW). Detailed instrumental conditions are provided in Table S6 (Supplementary Material). 

### 2.7. Quality Assurance and Quality Control (QA/QC)

Quality assurance and quality control (QA/QC) procedures were applied throughout sample preparation and ICP-MS analysis. Reagent and procedural blanks were included in each digestion batch to monitor contamination. Analytical precision was verified using three independent digestion replicates followed by separate ICP-MS measurements, while instrument stability was controlled using internal standards (Rh, In, Bi) and periodic recalibration. Method accuracy was assessed using certified reference materials (CRMs).

Digestion completeness was verified by the absence of visible residues after microwave digestion, clear final digest solutions, and consistent CRM recoveries (93–99%) across elements with different physicochemical behavior. Replicate digestion reproducibility remained below 5% RSD. Procedural blank concentrations were consistently very low or below the method detection limits for most analytes and did not significantly influence quantitative results.

Additional validation was performed for analytically challenging ultra-trace elements, particularly Sb and Sn, using the certified reference material BCR-129. Recoveries for Sb and Sn were 93.8% and 95.0%, respectively, with RSD values below 5%. Cr and Ni recoveries were 97.8% and 97.9%, respectively, confirming acceptable analytical accuracy and precision for interference-prone elements.

Multiple CRMs with botanical, geological, and mixed-environment matrices were used to validate the broad elemental range investigated in the present study. Potential matrix-related effects were minimized through microwave digestion, acid-matched calibration, He-KED collision mode, and internal standard correction. Recoveries for all investigated analytes ranged between 93% and 99%, with RSD values below 5%.

Limits of detection (LOD) and quantification (LOQ) were determined from replicate reagent and procedural blanks processed through the complete analytical workflow using the conventional 3σ and 10σ criteria, respectively. QA/QC acceptance criteria included recoveries between 90 and 110% and RSD ≤ 5%. Detailed analytical validation parameters are provided in the Supplementary Material (Tables S7–S9).

Analytical repeatability showed RSD values between 2.1% and 4.5%, while inter-batch reproducibility remained within 3–8% RSD. Instrument stability remained within ±3.2–4.8% for internal standards, and calibration verification standards analyzed every 10–15 samples showed deviations below 5%, indicating stable ICP-MS performance throughout the analytical sequences.

Measurement uncertainty was estimated using a simplified bottom–up approach based on the combined contribution of repeatability, calibration uncertainty, CRM recovery variability, and instrumental stability. Expanded uncertainty (U, k = 2, corresponding to approximately 95% confidence) was estimated for representative analytes. Estimated expanded uncertainty values were approximately 7.8% for K, 7.2% for Fe, 8.9% for Zn, 11.6% for Pb, 9.8% for Cd, 10.5% for As, and 12.3% for Hg. These uncertainty levels are consistent with the observed analytical precision (RSD < 5%), CRM recoveries (93–99%), and stable ICP-MS performance characteristics, supporting the reliability of concentration data and food safety interpretation. However, the reported uncertainty values should be interpreted as approximate analytical estimates intended to support data reliability assessment rather than as a complete metrological uncertainty budget according to full ISO/GUM methodology.

### 2.8. Data Processing and Statistical Analysis

All analytical results were expressed as mg/kg dry weight (d.w.). Different superscript letters within the same column indicate statistically significant differences between sampling sites or site categories at *p* < 0.05 according to the applied post hoc multiple-comparison tests (Tukey HSD or Games–Howell, as appropriate). Data processing was performed after verification of QA/QC criteria, including blank correction and validation against LOD and LOQ thresholds. Descriptive statistical analysis was applied to all datasets, and results are presented as mean values ± standard deviation (SD) based on triplicate measurements. Prior to inferential statistical analysis, data distribution normality was evaluated using the Shapiro–Wilk test, and homogeneity of variances was assessed using Levene’s test. When necessary, data were log10-transformed to improve normality and reduce heteroscedasticity. Statistical differences between sample groups were evaluated using one-way ANOVA. When ANOVA assumptions were met, Tukey’s honestly significant difference (HSD) test was applied for post hoc comparisons. When homogeneity of variance was not satisfied, Welch’s ANOVA followed by the Games–Howell post hoc test was used.

In addition to one-way ANOVA and Pearson correlations, advanced multivariate analyses were conducted to explore the structure of the multi-element dataset and evaluate spatial compositional patterns across sampling sites. Pearson correlation analysis was performed after verification of linearity and approximate normality of the variables. To control for multiple testing across the multi-element dataset, *p*-values were adjusted using the Benjamini–Hochberg false discovery rate (FDR) procedure where applicable. These included Principal Component Analysis (PCA), Hierarchical Cluster Analysis (HCA), heatmap visualization using standardized z-scores, and exploratory pattern-recognition approaches based on elemental composition. Prior to multivariate analysis, data were standardized using z-score normalization (mean-centering and unit-variance scaling) to eliminate the influence of different concentration ranges among elements. PCA was performed on the correlation matrix. HCA was conducted using Euclidean distance and Ward’s linkage method. Discriminant pattern-recognition approaches were used exclusively for exploratory visualization of group separation and were not validated as predictive classification models, due to the limited number of sites in certain categories and the associated risk of overfitting. These analyses were primarily intended as descriptive and comparative tools for multielement data interpretation and visualization rather than as direct quantitative proof of environmental contamination or causality. Because some pollution categories were represented by a limited number of sites, including one site for the polluted category, statistical comparisons among pollution categories were interpreted as exploratory and descriptive. Individual shrubs were treated as biological replicates within sites, while site-level differences were interpreted cautiously to avoid overgeneralization of pollution-category effects.

All statistical analyses were performed using IBM SPSS Statistics 29.0 (IBM Corp., Armonk, NY, USA) and XLSTAT 2025.5 (Addinsoft, Paris, France). Graphical outputs were generated and refined accordingly. A significance level of *p* < 0.05 was considered statistically significant.

### 2.9. Site Classification and Environmental Assessment

Sampling sites were classified according to pollution level and environmental pressure using a semi-quantitative multi-criteria framework integrating: (i) surrounding land use, (ii) proximity to potential contamination sources, (iii) distance to major roads and transport corridors, (iv) estimated agricultural intensity based on cultivated land and likely fertilizer/pesticide inputs, and (v) documented historical mining activity where relevant (Table S2). Spatial support for site characterization was derived from field observations, digital elevation data, topographic maps, and regional land-cover sources.

Based on the combined environmental evidence, sites were assigned to four categories: non-polluted, low impacted, moderately impacted, and polluted, representing increasing anthropogenic pressure. Non-polluted sites were remote or low-disturbance areas with minimal direct human influence, whereas polluted sites were associated with documented anthropogenic legacy sources, particularly historical mining activity.

To support comparative interpretation, the qualitative site classification was additionally examined in relation to integrated contamination indices derived from fruit elemental composition (Section 2.10). These indices were used as complementary exploratory tools for evaluating multielement accumulation patterns across sites rather than as independent quantitative measures of environmental contamination. This framework provided a consistent basis for comparative assessment of spatial variation in elemental accumulation in *Prunus spinosa* fruits.

### 2.10. Integrated Contamination Indices and Multi-Element Assessment

To assess relative multi-element variation across sampling sites, the contamination factor (CF) was calculated for each analyzed element. CF is defined as the ratio between the element concentration in the sample and its corresponding baseline concentration, according to the following equation:

$$CF_{i}=\frac{C_{i}}{C_{background}}$$

where $C_{i}$ is the concentration of element *i* in the sample (mg/kg dry weight), and $C_{background}$ is the reference baseline concentration. In this study, $C_{background}$ was defined as the mean concentration of each element measured in samples from non-polluted sites. These sites were selected based on the environmental classification described in Section 2.9 and Table S2, representing areas with minimal anthropogenic influence. This approach ensures consistency with the study design and provides a local comparative reference for evaluating relative elemental enrichment patterns across sampling sites rather than an absolute geochemical background value.

To obtain an integrated comparative assessment across sampling sites, the Pollution Load Index (PLI) was calculated using the contamination factors of the analyzed elements. PLI was determined as the geometric mean of the individual contamination factors, according to the following equation:

$$PLI={\left(CF_{1}\times CF_{2}\times \cdots \times CF_{n}\right)}^{1/n}$$

where $CF_{1},CF_{2},\ldots ,CF_{n}$ represent the contamination factors of the individual elements, and n is the total number of analyzed elements included in the calculation. The Pollution Load Index was used as an exploratory comparative indicator of relative multi-element enrichment among sampling sites with different environmental conditions and degrees of anthropogenic influence.

In addition, the Metal Pollution Index (MPI) was calculated to assess the overall metal load in the analyzed samples. The MPI was determined as the geometric mean of the concentrations of the selected elements, according to the following expression:

$$MPI={\left(C_{1}\times C_{2}\times \cdots \times C_{n}\right)}^{1/n}$$

where $C_{1},C_{2},\ldots ,C_{n}$ represent the concentrations of the analyzed elements (mg/kg dry weight), and *n* is the number of elements included in the calculation.

The Metal Pollution Index provided a complementary measure of cumulative elemental burden and was primarily intended for comparative evaluation of spatial accumulation patterns across sampling sites.

Furthermore, the Toxic Composite Index (TCI) was calculated to provide an integrated comparative assessment of toxic-element variability across sampling sites. The TCI was calculated as the mean standardized z-score of the analyzed toxic and contaminant-related elements (Pb, Cd, As, Hg, Ni, Cr, Sb, and Sn), according to the following expression:

TCI = (z_Pb_ + z_Cd_ + z_As_ + z_Hg_ + z_Ni_ + z_Cr_ + z_Sb_ + z_Sn_)/n

where z represents the standardized z-score value of each element and n is the total number of toxic and contaminant-related elements included in the calculation. The TCI was used as an exploratory comparative indicator of relative toxic-element enrichment across sampling sites rather than as an absolute quantitative measure of environmental contamination.

Because all indices were derived from the same elemental dataset, they should be interpreted as complementary exploratory tools rather than independent quantitative measures of environmental contamination.

### 2.11. Human Exposure and Health Risk Assessment

The potential human exposure to trace elements through the consumption of *Prunus spinosa* fruits was evaluated by calculating the estimated daily intake (EDI) and the hazard quotient (HQ). The EDI was calculated using the following equation:

$$EDI=\frac{C\times IR}{BW}$$

where *C* represents the concentration of the element in the fruit (mg/kg fresh weight), *IR* is the daily ingestion rate (kg/day), and *BW* is the average body weight (kg). The non-carcinogenic health risk associated with metal intake was assessed using the hazard quotient (HQ), calculated as:

$$HQ=\frac{EDI}{RfD}$$

where *RfD* is the oral reference dose for each element (mg/kg/day), obtained from international regulatory agencies. For the exposure assessment, an average adult body weight of 70 kg was assumed. The daily ingestion rate of fruits was considered based on literature values for fruit consumption. Reference dose (RfD) values were set at 0.0035 mg/kg/day for Pb and 0.001 mg/kg/day for Cd, based on guidelines provided by the United States Environmental Protection Agency and international risk assessment frameworks.

The present exposure assessment was based on total elemental concentrations determined by ICP-MS and therefore represents a preliminary screening-level evaluation. Elemental speciation, gastrointestinal bioaccessibility, and inter-individual dietary exposure variability were not investigated in the current study. Consequently, the calculated EDI and HQ values should be interpreted as comparative exposure indicators rather than definitive toxicological risk estimates.

### 2.12. Ethical Statement

This study did not involve human participants or vertebrate animals. The investigated plant material (*Prunus spinosa* L.) was collected from natural populations, and no endangered or protected species were affected. Therefore, no specific ethical approval was required for this research.

### 2.13. Data Availability Statement

All relevant data supporting the findings of this study are included within the article and its Supplementary Material. Additional data are available from the corresponding author upon reasonable request.

## 3. Results

### 3.1. Overview of Elemental Composition in Prunus spinosa Fruits

The elemental composition of *Prunus spinosa* fruits varied across the sampling sites and pollution categories, revealing distinct patterns among macroelements, essential microelements, geochemical trace elements, and contaminant-related elements. Macroelements such as K, Ca, and Mg generally showed higher concentrations in fruits collected from non-polluted sites, whereas Na tended to increase toward the more impacted categories. Among essential microelements, Fe, Mn, Zn, Cu, Co, and Se displayed increasing concentrations along the site classification gradient, while B and Mo showed the opposite tendency. Geochemical trace elements, including Al, Si, Ba, Sr, Li, Rb, Cs, V, and Ti, were consistently lower in non-polluted sites and higher in moderately impacted and polluted sites. A similar gradient was observed for toxic and potentially toxic elements, including Pb, Cd, As, Hg, Ni, Cr, Sb, and Sn, whose concentrations increased progressively from non-polluted to more impacted categories. The elevated concentrations observed for Sb, Sn, Cr, and partly Ni were not randomly distributed across samples but generally increased toward traffic-influenced, agricultural, and mining-affected environments, particularly at ZLA-04 and EG-10. This spatial distribution pattern is consistent with a potential environmental contribution associated with anthropogenic pressure rather than analytical artefacts. Furthermore, the analytical reliability of these elements was supported by He-KED interference reduction, optimized isotope selection, CRM validation, low RSD values, and stable internal standard recoveries throughout the analytical sequences.

In contrast, S remained relatively stable across all sampling sites. Overall, the results indicate a gradual change in the multielement profile of the fruits across the pollution gradient, with distinct differences among site categories.

### 3.2. Distribution of Macroelements Across Sampling Sites

Potassium (K), calcium (Ca), and magnesium (Mg) display similar distribution patterns across sampling sites, with generally higher concentrations in sites classified as non-polluted and lower values in the low impacted, moderately impacted, and polluted site categories (Table 2). Potassium ranges from 16,868 ± 360 mg/kg in polluted sites to 19,028 ± 405 mg/kg in non-polluted areas, calcium from 1365 ± 57 mg/kg in moderately impacted sites to 1562 ± 48 mg/kg in non-polluted sites, and magnesium from 871 ± 31 mg/kg in moderately impacted sites to 991 ± 55 mg/kg in non-polluted areas. All three elements show statistically significant differences among site categories (*p* < 0.05), indicating a decreasing trend across site categories arranged along the pollution gradient.

Phosphorus (P) exhibits a less consistent distribution across site categories, ranging from 1411 ± 70 mg/kg in polluted areas to 1629 ± 135 mg/kg in moderately impacted sites. Elevated concentrations are observed in both low- and moderately impacted sites, without a clear monotonic relationship with site classification level, although differences remain statistically significant.

Sodium (Na) shows an increasing trend across the site classification gradient, with concentrations rising from 497 ± 38 mg/kg in non-polluted sites to 721 ± 85 mg/kg in polluted areas (*p* < 0.05).

Sulfur (S) exhibited relatively constant values across all sampling sites, ranging from 4925.51 ± 60.00 mg/kg to 5026.77 ± 64.80 mg/kg DW, with all samples belonging to the same statistical group and no significant differences observed among site categories (*p* > 0.05).

These patterns are further supported by the integrated dataset (Table S11), which highlights a consistent decreasing trend for K, Ca, and Mg across sites classified along the pollution gradient, alongside a marked increase in Na (+41.1% compared with non-polluted sites). The stability of S and the relatively constant Ca/Mg and K/Ca ratios suggest limited variation in major-element balance across site categories, while the decline in the K/Na ratio suggests a shift in the relative proportion of these elements across sites assigned to higher-impact categories.

### 3.3. Profile of Essential Microelements in Prunus spinosa

The essential microelements in *Prunus spinosa* fruits exhibit two distinct distribution patterns across the site classification gradient: (i) a group of elements showing progressive increases in concentration (Fe, Mn, Zn, Cu, Co, and Se), and (ii) elements displaying decreasing trends (B and Mo) (Table 3).

Iron (Fe), manganese (Mn), zinc (Zn), copper (Cu), cobalt (Co), and selenium (Se) show progressively higher concentrations across site categories, from non-polluted to polluted sites. Fe concentrations range from 17.9–20.3 mg/kg in non-polluted sites (PON-03, RAM-02, GAR-07) to 38.9 mg/kg in the polluted site (ZLA-04), with intermediate values in low- and moderately impacted areas. A similar pattern is observed for Mn, increasing from 3.5–4.2 mg/kg to 9.8 mg/kg, Zn from 5.9–6.8 mg/kg to 14.8 mg/kg, and Cu from 2.0–2.3 mg/kg to 4.8 mg/kg along the same gradient. Cobalt and selenium follow comparable trends, with Co rising from 0.11–0.13 mg/kg in non-polluted sites to 0.25 mg/kg in polluted areas, and Se increasing from 0.024–0.026 mg/kg to 0.038 mg/kg. These increases are statistically significant (*p* < 0.05) and are consistent with higher concentrations observed in the investigated sites classified as more impacted.

In contrast, boron (B) and molybdenum (Mo) show decreasing concentrations across sites classified as more impacted. B concentrations decline from 17.2–18.5 mg/kg in non-polluted sites to 13.1 mg/kg in polluted areas, while Mo decreases from 0.40 mg/kg to 0.25 mg/kg across the same site categories. Although the differences among impacted categories are less pronounced for Mo, both elements show statistically significant variations (*p* < 0.05), suggesting differences in availability and/or accumulation patterns across sites assigned to higher-impact categories.

The integrated analysis (Table S12) reinforces these observations, showing substantial increases in Fe (+105.8%), Mn (+157.9%), Zn (+134.9%), and Cu (+128.6%) in the more impacted site categories. Conversely, B and Mo exhibit pronounced declines (−26.4% and −34.2%, respectively). Elemental ratios such as B/Fe and Mo/Fe decrease sharply, suggesting a shift in the relative distribution of these elements across site categories.

### 3.4. Geochemical Trace Element Signature of the Samples

The concentrations of trace elements (Al, Si, Ba, Sr, Li, Rb, Cs, V, and Ti) in *Prunus spinosa* fruits are progressively higher across the site classification gradient, from non-polluted site categories to more impacted site categories, with statistically significant differences (*p* < 0.05) (Table 4).

It should be noted that all fruit samples were washed with distilled water before analysis to reduce superficial contamination and improve sample comparability. Consequently, part of the loosely deposited particulate fraction may have been removed during sample preparation. Therefore, the measured concentrations likely reflect a combination of internal elemental uptake and more strongly retained surface-associated particles rather than total atmospheric deposition alone. As a result, interpretations regarding dust and atmospheric particulate influence should be considered cautiously, particularly for lithogenic elements such as Al and Si.

Sites classified as non-polluted (RAM-02, PON-03, GAR-07) show the lowest values for all analyzed elements, whereas low- and moderately impacted sites exhibit intermediate levels. The highest concentrations are observed in the more impacted sites, particularly NOJ-08, EG-10, and ZLA-04.

For example, Al and Si range from 11.76–13.28 mg/kg and 43.68–47.61 mg/kg in non-polluted sites to 30.84–36.58 mg/kg and 78.15–88.73 mg/kg in moderately impacted and polluted sites, respectively. Similar increasing patterns are recorded for Ba (1.71–1.94 to 4.12–4.83 mg/kg) and Sr (2.96–3.36 to 7.08–8.36 mg/kg).

Li, Rb, and Cs also display a gradual increase, from 0.019–0.022 mg/kg to 0.046–0.053 mg/kg (Li), 2.18–2.52 mg/kg to 5.18–6.07 mg/kg (Rb), and 0.016–0.018 mg/kg to 0.029–0.034 mg/kg (Cs). Similarly, V and Ti increase from 0.171–0.194 mg/kg to 0.403–0.482 mg/kg and from 0.81–0.89 mg/kg to 1.71–1.94 mg/kg, respectively.

These trends are consistently reflected in the integrated geochemical indicators (Table S13), where all trace elements show significant increases, particularly Al (+192.8%) and V (+164.8%). The progressive increase in Al/Si and V/Ti ratios suggests greater geochemical differentiation across the site classification framework, while the relative stability of Ba/Sr and Rb/Sr ratios indicates limited variation in these ratios among site categories.

### 3.5. Heavy Metals and Contaminant-Related Elements

To ensure methodological consistency, concentrations expressed on a dry weight (DW) basis were converted to wet weight (WW) using sample-specific moisture content (78–83%), according to the equation C_WW = C_DW × (1 − f), as detailed in Table S14.

#### 3.5.1. Toxic Elements (Pb, Cd, As, Hg)

Lead (Pb) concentrations in *Prunus spinosa* fruits range from 0.019 ± 0.003 mg/kg (PON-03, non-polluted site) to 0.052 ± 0.009 mg/kg (ZLA-04, site classified as polluted). Lower values are characteristic of sites classified as non-polluted (0.019–0.022 mg/kg), whereas progressively higher concentrations are observed across increasingly impacted site categories, with the highest values recorded at sites classified as polluted (NOJ-08: 0.038 ± 0.007 mg/kg; EG-10: 0.044 ± 0.008 mg/kg; ZLA-04: 0.052 ± 0.009 mg/kg). Differences among site categories are statistically significant (*p* < 0.05) (Table 5).

Cadmium (Cd) values vary between 0.016 ± 0.002 mg/kg (PON-03, non-polluted) and 0.036 ± 0.006 mg/kg (ZLA-04, polluted). Non-polluted sites show the lowest concentrations (0.016–0.018 mg/kg), whereas a clear increasing trend is observed across sites classified along the pollution gradient, with the highest values recorded in moderately impacted and polluted sites, with statistically significant differences among site categories (*p* < 0.05).

Arsenic (As) concentrations range from 0.171 ± 0.019 mg/kg (PON-03, non-polluted) to 0.421 ± 0.045 mg/kg (ZLA-04, polluted). Similar to Pb and Cd, lower values are recorded in non-polluted sites (0.171–0.194 mg/kg), while elevated concentrations occur in the more impacted site categories, particularly in sites classified as moderately impacted or polluted (NOJ-08: 0.331 ± 0.036 mg/kg; EG-10: 0.368 ± 0.040 mg/kg; ZLA-04: 0.421 ± 0.045 mg/kg), with statistically significant differences.

Mercury (Hg) varies between 0.018 ± 0.003 mg/kg (PON-03, non-polluted) and 0.039 ± 0.007 mg/kg (ZLA-04, polluted). A gradual increase is observed from non-polluted sites (0.018–0.020 mg/kg) to low and moderately impacted sites, reaching the highest concentrations in polluted areas, indicating statistically significant differences among site categories.

In addition, the integrated contaminant profile (Table S15) shows substantially higher concentrations of toxic elements, including Pb (+147.6%), Cd (+111.8%), As (+131.3%), and Cr (+148.6%), in the more impacted site categories. The relative stability of ratios such as Ni/Cr indicates limited variation across site categories, while increases in the Pb/Cd and As/Hg ratios reflect differences in the relative proportions of these elements among site categories.

The conversion to wet weight (Table S14) provides a more relevant basis for assessing potential dietary exposure through fruit consumption, while the regulatory interpretation framework and analytical comparability of the investigated elements are summarized in Table S10 (Supplementary Material). Although Pb and Cd concentrations are higher in sites assigned to the more impacted categories, their wet weight values remain substantially lower due to the high moisture content of the fruits (78–83%). This underscores the importance of accounting for matrix-specific water content when evaluating compliance with regulatory thresholds.

#### 3.5.2. Potentially Toxic Elements (Ni, Cr)

Nickel (Ni) concentrations range from 1.71 ± 0.19 mg/kg (PON-03, non-polluted) to 4.47 ± 0.52 mg/kg (ZLA-04, site classified as polluted). Lower values are associated with non-polluted sites (1.71–1.94 mg/kg), while progressively higher concentrations are observed across the site classification gradient, with maximum concentrations in moderately impacted and polluted sites (NOJ-08: 3.36 ± 0.38 mg/kg; EG-10: 3.82 ± 0.44 mg/kg; ZLA-04: 4.47 ± 0.52 mg/kg), indicating statistically significant differences (*p* < 0.05) (Table 6).

Chromium (Cr) values vary between 2.96 ± 0.33 mg/kg (PON-03, non-polluted) and 7.88 ± 0.94 mg/kg (ZLA-04, polluted). Non-polluted sites exhibit the lowest concentrations (2.96–3.36 mg/kg), while impacted sites show progressively higher values, with the highest values recorded in the more strongly impacted site categories (NOJ-08: 5.94 ± 0.72 mg/kg; EG-10: 6.71 ± 0.81 mg/kg; ZLA-04: 7.88 ± 0.94 mg/kg), with statistically significant differences.

#### 3.5.3. Additional Contaminant-Related Elements (Sb, Sn)

Antimony (Sb) concentrations range from 2.18 ± 0.27 mg/kg (PON-03, non-polluted) to 5.88 ± 0.72 mg/kg (ZLA-04, site classified as polluted). Lower values are characteristic of non-polluted sites (2.18–2.52 mg/kg), whereas progressively higher concentrations are observed across the low impacted, moderately impacted, and polluted site categories, with maximum values recorded at sites classified as polluted (NOJ-08: 4.46 ± 0.55 mg/kg; EG-10: 5.02 ± 0.61 mg/kg; ZLA-04: 5.88 ± 0.72 mg/kg), with statistically significant differences (*p* < 0.05) (Table 7).

Tin (Sn) values vary between 11.76 ± 1.34 mg/kg (PON-03, non-polluted) and 36.58 ± 4.18 mg/kg (ZLA-04, polluted). Non-polluted sites show the lowest concentrations (11.76–13.28 mg/kg), whereas higher values are observed in the more impacted site categories, with the highest values recorded in moderately impacted and polluted sites, indicating statistically significant differences.

### 3.6. Spatial Variation in Elemental Composition Across Environmental Gradients

#### 3.6.1. Trends in Major Elements

**Figure 1:**
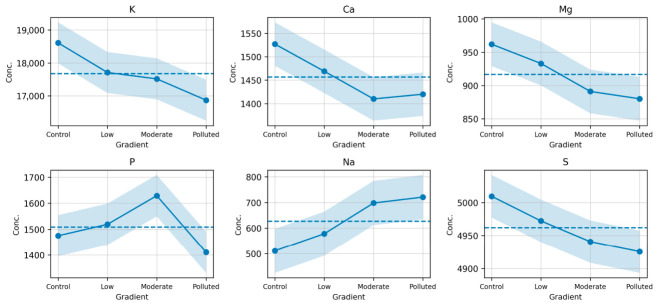
Variation in Macroelement Concentrations in *Prunus spinosa* Fruits the Pollution Gradient.

The concentrations of major elements in *Prunus spinosa* fruits varied consistently across the site classification gradient (non-polluted → low impacted → moderately impacted → polluted) (Figure 1). Potassium (K), calcium (Ca), and magnesium (Mg) decreased progressively, whereas sodium (Na) increased. Phosphorus (P) showed a non-linear pattern, with slightly higher values at low- and moderately impacted sites followed by a decline in the polluted category. Sulfur (S) concentrations remained relatively stable across all site categories.

Concentrations of trace elements exhibited site-dependent variation across the site classification framework. Iron (Fe) and manganese (Mn) showed higher values in fruits collected from sites classified as moderately impacted and polluted. Zinc (Zn) and copper (Cu) varied without a consistent linear trend across the gradient. Lead (Pb) and cadmium (Cd) concentrations were higher in samples collected from polluted sites. Overall, elemental concentrations varied consistently across the site classification framework.

#### 3.6.2. Behavior of Micronutrients Along the Gradient

**Figure 2:**
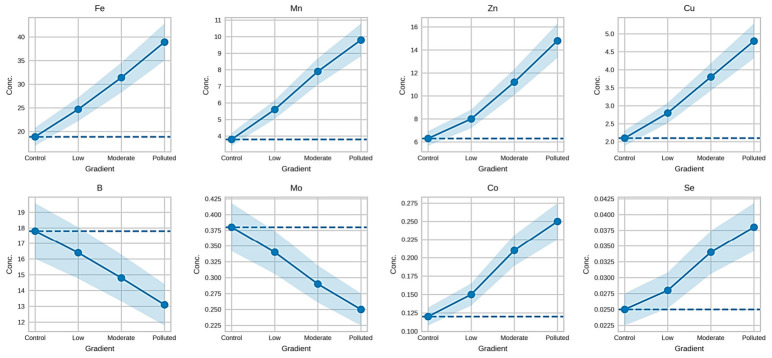
Variation in Essential Microelement Concentrations in *Prunus spinosa* Fruits Along the Pollution Gradient.

The concentrations of essential micronutrients in *Prunus spinosa* fruits varied across sites classified in the pollution gradient (Figure 2). Iron (Fe), manganese (Mn), zinc (Zn), and copper (Cu) showed a progressive increase, a pattern also observed for cobalt (Co) and selenium (Se), albeit at lower absolute concentrations. In contrast, boron (B) and molybdenum (Mo) decreased across sites classified in the pollution gradient.

#### 3.6.3. Distribution Patterns of Trace and Toxic Elements

**Figure 3:**
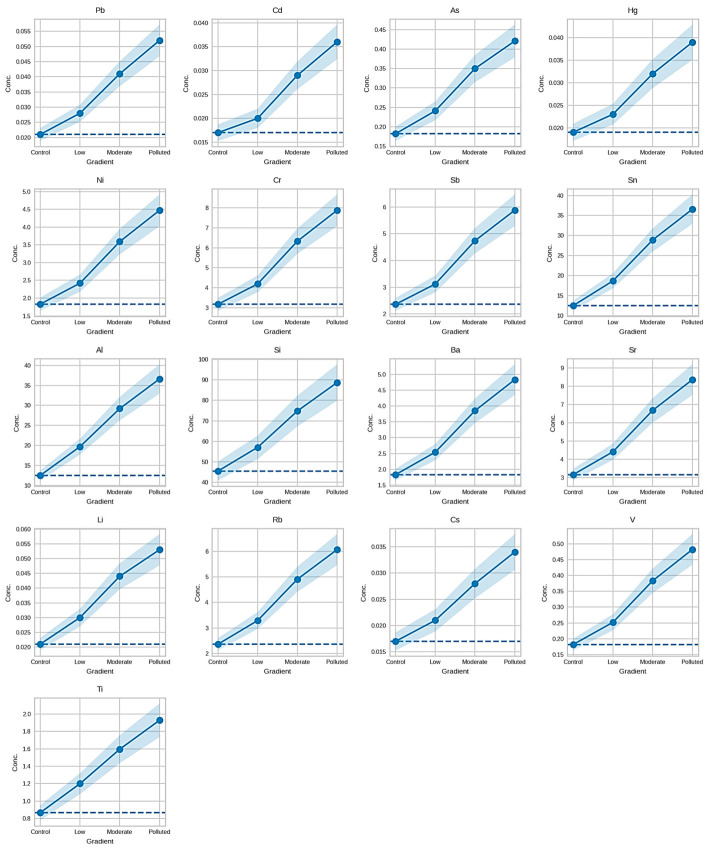
Variation in Toxic and Trace Element Concentrations in *Prunus spinosa* Fruits Along the Pollution Gradient.

Trace and potentially toxic elements in *Prunus spinosa* fruits showed progressively higher values across sites classified along the pollution gradient (Figure 3). This trend was observed for Pb, Cd, As, Hg, Ni, Cr, Sb, and Sn, and extended to geochemical-associated elements, including Al, Si, Ba, Sr, Li, Rb, Cs, V, and Ti.

### 3.7. Multivariate Analysis of Elemental Profiles

#### 3.7.1. Heatmap Analysis of Standardized Elemental Profiles

**Figure 4:**
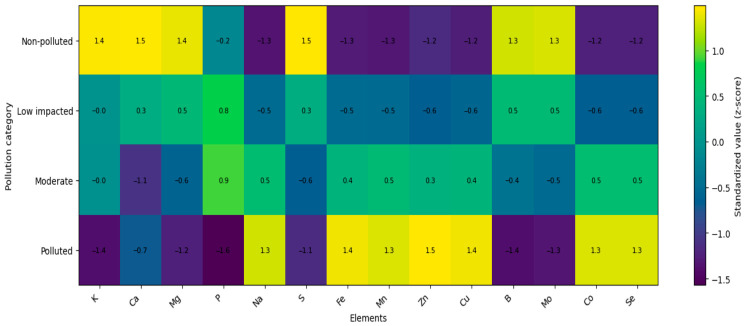
Heatmap of standardized macro- and micronutrient profiles.

**Figure 5:**
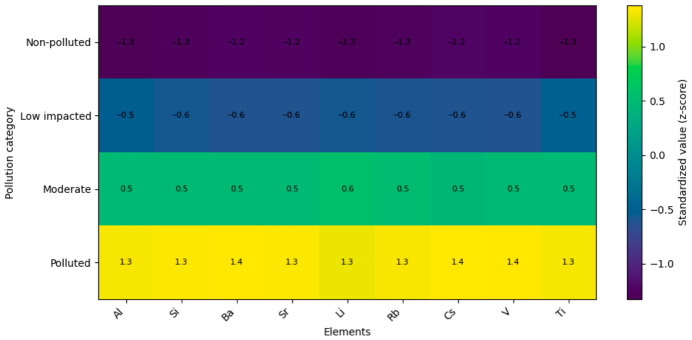
Heatmap of standardized geochemical element concentrations.

**Figure 6:**
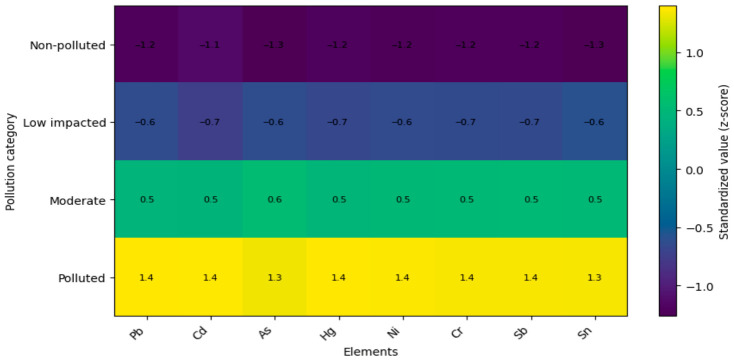
Heatmap of standardized toxic element concentrations.

The standardized elemental profiles (z-scores) revealed a consistent separation among site categories across all element groups (Figure 4, Figure 5 and Figure 6). Non-polluted sites were characterized by predominantly negative standardized values for most trace, geochemical, and toxic elements, whereas several macroelements and selected micronutrients exhibited positive deviations.

Low impacted sites displayed intermediate values, typically close to zero, indicating a transitional composition. In moderately impacted sites, a shift toward positive values became evident for several elements, particularly among micronutrients and geochemical elements (Figure 4 and Figure 5).

Sites classified as polluted were characterized by strongly positive standardized values across most trace, geochemical, and toxic elements (e.g., Pb, Cd, As, Hg, Ni, Cr, Sb, Sn, Al, Si, Ba, Sr, Li, Rb, Cs, V, Ti), whereas macroelements such as K, Ca, and Mg showed negative deviations (Figure 5 and Figure 6).

#### 3.7.2. Principal Component Analysis (PCA)

Principal component analysis (PCA) further confirmed the separation of elemental patterns across site categories, with PC1 representing the dominant axis of variation (Table 8). Loadings on PC1 ranged from −0.305 to 0.308, indicating a strong contrast between two groups of elements with opposing patterns.

Elements with negative loadings on PC1, including B (−0.305), Mo (−0.305), K (−0.250), Ca (−0.234), and Mg (−0.221), were associated with lower values in the more impacted site categories. The relatively high negative loadings of B and Mo (−0.305) indicate a strong contribution to this axis. Similarly, Ca (−0.234), Mg (−0.221), and K (−0.250) also contributed to the negative side of PC1, reflecting a coherent pattern among these elements.

In contrast, elements with positive loadings on PC1—Al (0.308), Mn (0.307), Cu (0.306), Fe (0.306), Zn (0.304), Si (0.300), and Na (0.299)—displayed an increasing trend across sites classified along the pollution gradient. The relatively uniform loadings (approximately 0.30) indicate a similar contribution of these elements to the variance explained by PC1, suggesting a consistent pattern across this group.

PC2, with loadings ranging from −0.543 to 0.682, represents a secondary axis of variation. Mg exhibited the highest positive loading on PC2 (0.682), while Ca showed a strong negative loading (−0.543), indicating that these elements are key contributors to this axis. Other elements displayed relatively low loadings on PC2 (e.g., Fe = 0.111, Zn = 0.114, Mo = 0.095), suggesting a more limited influence on this component. Both heatmap visualization and PCA consistently supported the differentiation of sampling sites according to pollution intensity and multi-element composition.

#### 3.7.3. Discriminant Analysis Using All Quantified Elements

**Figure 7:**
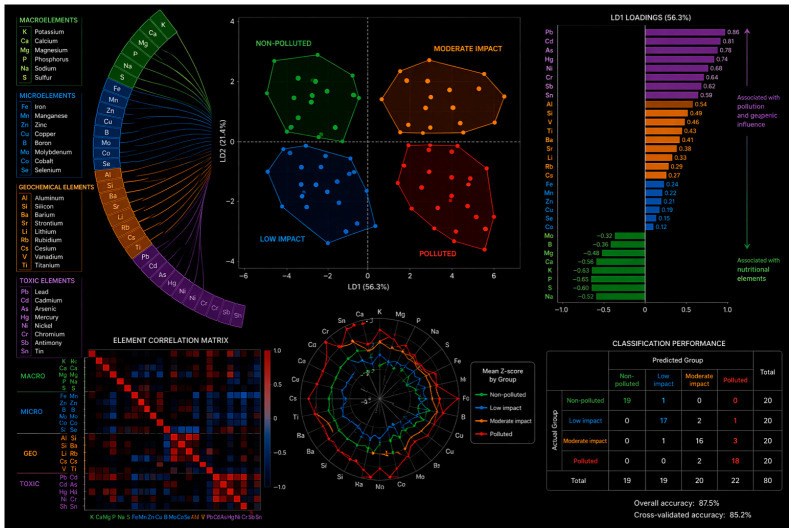
Multivariate discriminant fingerprinting of macroelements, micronutrients, geochemical tracers, and toxic elements in *Prunus spinosa* fruits along the pollution gradient.

The integrated chemometric analysis clearly shows that the complete elemental profile of *Prunus spinosa* fruits changes consistently along the pollution gradient. Samples from non-polluted sites were distinctly separated from those collected in moderately impacted and polluted areas, indicating that environmental pressure strongly influences fruit mineral composition (Figure 7).

The main discriminating variables were contaminant-related elements (Pb, Cd, As, Hg, Ni, Cr, Sb, Sn) together with geochemical tracers (Al, Si, V, Ti), which were associated with impacted sites, whereas nutritionally important elements such as K, Ca, Mg, B, and Mo were linked to non-polluted areas. This suggests a progressive shift from nutrient-dominated to contamination-influenced elemental signatures.

Importantly, the exploratory multivariate analysis suggests that *Prunus spinosa* fruits may have potential as bioindicators of habitat quality when the integrated multielement fingerprint is considered rather than individual toxic metals alone. Thus, Figure 7 provides exploratory chemometric evidence supporting the potential applicability of wild edible fruits in environmental biomonitoring.

### 3.8. Integrated Multi-Metal Contamination Assessment Using CF, PLI, MPI and Composite Toxicity Indices

**Figure 8:**
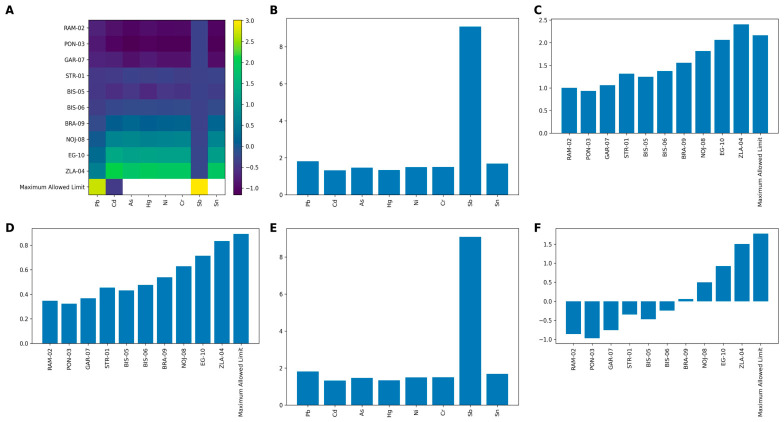
Integrated multi-metal contamination assessment across study sites based on standardized profiles and composite indices. (**A**) Heatmap of normalized metal concentrations (z-score) for Pb, Cd, As, Hg, Ni, Cr, Sb and Sn across sampling sites, including the maximum allowed limits for comparison. (**B**) Contamination Factor (CF) for individual metals relative to the internal baseline (RAM-02, PON-03, GAR-07). (**C**) Pollution Load Index (PLI) showing the cumulative contamination level at each site. (**D**) Metal Pollution Index (MPI) reflecting the overall multi-element concentration burden. (**E**) Fold-change (enrichment ratio) of metals relative to the baseline. (**F**) Toxic Composite Index (TCI), calculated as the mean z-score of all analyzed metals, illustrating the integrated toxicity gradient across sites.

The integrated analysis of multi-metal contamination revealed a clear and consistent pattern across the investigated sites, as evidenced by all applied indices (Figure 8). The heatmap of standardized concentrations (Figure 8A) showed that reference sites (RAM-02, PON-03 and GAR-07) were characterized by predominantly negative z-score values, whereas sites assigned to the impacted categories displayed progressively higher standardized levels, with the highest values observed at ZLA-04. The most pronounced increases were observed for Sn, Cr and Sb, which consistently exhibited elevated standardized values across the most impacted locations.

The Contamination Factor (Figure 8B) indicated moderate enrichment for most elements. However, Sb showed a markedly elevated CF, clearly identifying it as the element with the highest relative enrichment in the dataset. Pb and Sn also displayed relatively higher CF values, whereas Cd and Hg remained closer to baseline conditions.

At the site level, the Pollution Load Index (Figure 8C) demonstrated a gradual increase from reference to impacted environments. Reference sites showed PLI values close to unity, whereas impacted sites reached the highest values at ZLA-04, indicating higher cumulative metal levels relative to the internal baseline. All impacted sites exhibited PLI values above 1, indicating deviation from the internal baseline conditions. The Metal Pollution Index (Figure 8D) showed a similar increasing trend, with the lowest values observed at reference sites and the highest at the most impacted locations. This pattern is consistent with higher multi-element concentrations and closely mirrors the PLI-based ranking of sites. Fold-change analysis (Figure 8E) further highlighted the stronger relative enrichment of specific elements. Relative to baseline conditions, Sb showed the strongest enrichment, while Sn, Cr and Pb also exhibited marked increases. In contrast, Cd and Hg displayed only minor deviations from baseline. The Toxic Composite Index (Figure 8F) showed a continuous transition from negative values at reference sites to strongly positive values at impacted sites, with the maximum observed at ZLA-04. This pattern is consistent with progressively higher combined toxic-element levels across the site categories and highlights the ability of TCI to integrate multi-element variability into a single descriptor.

## 4. Discussion

### 4.1. Interpretation of Elemental Patterns Along the Pollution Gradient

The elemental composition of *Prunus spinosa* fruits varied across sites classified along the environmental pressure gradient, indicating coupled effects of soil geochemistry and plant physiological responses under anthropogenic pressure.

The decline in essential macroelements (K, Ca, and Mg), together with increased Fe, Mn, Zn, and Cu concentrations, may be associated with altered nutrient acquisition processes under anthropogenic pressure. Previous studies have shown that soil acidification and reduced cation exchange capacity can influence the availability of base cations while increasing the mobility of trace metals in contaminated environments. In parallel, elevated divalent metals may contribute to competitive interactions affecting Ca and Mg uptake at root transport sites, potentially contributing to nutrient depletion.

Similar elemental patterns have previously been associated with physiological stress responses under metal exposure conditions, including potential alterations in membrane integrity and transporter selectivity. In the present study, the higher Na concentrations and lower K/Na ratios observed in impacted sites may be consistent with disturbed ionic balance under environmental stress conditions. In addition, non-specific transport pathways described in the literature (e.g., ZIP, NRAMP, and IRT families) could contribute to increased Fe, Zn, and Mn accumulation, although these mechanisms were not directly investigated in the present study.

The contrasting decrease in B and Mo suggests that pollution does not uniformly increase all elements, but rather reshapes micronutrient balance according to element-specific mobility and uptake constraints. Overall, the fruit ionome (total elemental composition of a biological organism or tissue) appears to reflect local environmental conditions and potential differences in element availability rather than total elemental abundance.

Biologically, these shifts may be associated with increased physiological adjustment requirements for maintaining nutrient balance under environmental stress conditions, whereas ecologically they may reflect alterations in normal soil–plant buffering processes. Consequently, fruit elemental composition functions as an integrated signal of both external contamination pressure and internal physiological response, supporting the use of *Prunus spinosa* as a bioindicator species.

### 4.2. Influence of Anthropogenic Sources on Elemental Accumulation

The spatial distribution of elemental concentrations in *Prunus spinosa* fruits is consistent with the influence of site-specific anthropogenic inputs and associated changes in local soil properties. Differences among sampling sites may also be influenced by unmeasured soil properties, including pH, redox conditions, and organic matter content [57,58].

The highest concentrations observed in the Zlatna area (ZLA-04) are consistent with legacy mining influence. Residual tailings, historically contaminated soils, and weathering of mining wastes may continue to release trace elements into surrounding environments. Processes described in mining-affected environments, including dissolution of Fe/Mn oxides under changing redox conditions, may contribute to metal mobility [59,60]. Under these conditions, elevated fruit concentrations could also be associated with altered plant uptake behavior under environmental stress conditions [61].

At the Bucea site (EG-10), elevated Fe and Zn concentrations are compatible with traffic-related inputs. Metals derived from brake wear, tire abrasion, vehicle corrosion, and resuspended roadside dust can accumulate in adjacent soils and on plant surfaces, contributing to plant exposure through both root uptake and direct deposition pathways [62,63].

At the agricultural site (Leș, NOJ-08), moderate enrichment may reflect long-term agrochemical inputs, including fertilizer application and pesticide residues. Repeated agricultural use can modify soil chemistry through acidification, complexation reactions, and altered cation exchange behavior, thereby increasing the mobility and plant availability of selected elements [64,65].

In contrast, non-impacted sites (GAR-07 and PON-03) showed lower elemental concentrations, consistent with background geochemical conditions and more stable soil–plant interactions. In such environments, elemental uptake is likely more strongly regulated by natural soil properties and plant physiological selectivity than by chronic anthropogenic disturbance [66].

Overall, the results suggest that elemental accumulation in *Prunus spinosa* fruits may be influenced by the combined effects of contamination source type and soil–plant transfer processes. Mining, traffic, and agricultural activities appear to generate partially distinct geochemical signatures that are subsequently filtered through local soil conditions and plant uptake mechanisms [67].

### 4.3. Bioindicator Potential of Prunus spinosa

The consistent variation in elemental composition across contrasting sites supports the potential use of *Prunus spinosa* as a bioindicator species in heterogeneous environments. The observed fruit chemistry appears to integrate multiple exposure pathways, including root uptake from soils and atmospheric particle deposition.

The simultaneous increase in several trace elements (Fe, Mn, Zn, Cu, Co, and Se) together with the decline of selected nutrients such as B and Mo suggests sensitivity not only to contamination intensity, but also to associated ecological and physiological stress. This bidirectional response indicates that the species may reflect both external environmental pressure and internal adjustments in nutrient regulation.

Importantly, the consistency of these elemental patterns across different pollution contexts suggests that *Prunus spinosa* responds to multi-source contamination in a coherent and measurable manner. Such integrated responses may provide more informative signals than single-element indicators alone.

Together, these traits—sensitivity, consistency, ecological adaptability, and multi-element accumulation behavior—support the consideration of *Prunus spinosa* as a promising bioindicator species for environmental monitoring in mixed-impact landscapes.

### 4.4. Comparison with Previous Studies

The macroelement composition of *Prunus spinosa* fruits is largely consistent with literature data, suggesting a relatively stable mineral profile across environments. Potassium (16,868–19,028 mg/kg DW) remains the dominant macroelement, while calcium, magnesium, and phosphorus fall within reported ranges by Marakoğlu et al. (2005) [68] suggesting strong physiological regulation. In contrast, slightly elevated sodium levels (497–721 mg/kg), particularly in impacted areas, may reflect reduced ionic selectivity under environmental stress. Sulfur concentrations remained relatively stable across sampling sites and were within the concentration range typically reported for plant tissues and wild fruits in the literature, suggesting a moderate influence of local environmental conditions [69]. Therefore, deviations from literature ranges likely reflect local geochemical context rather than species-specific anomalies.

Microelement concentrations show greater variability. Iron (17.9–38.9 mg/kg), zinc (5.9–14.8 mg/kg), and copper (2.0–4.8 mg/kg) exceed commonly reported ranges, approaching the upper literature values reported by Vlaicu et al. 2026 [70], especially in polluted samples. This pattern may suggest enhanced metal bioavailability, likely driven by soil processes (e.g., acidification, redox changes) and non-specific uptake mechanisms under stress. Manganese shows moderate variation, while boron and molybdenum remain relatively stable or decrease, suggesting stronger physiological control. Trace elements such as Co and Se occur at low levels but increase slightly in impacted environments. This variability indicates that microelements are more environmentally responsive than macroelements, which appear under stronger physiological control.

Aluminium, barium, and vanadium concentrations fall within the ranges reported by Jáudenes-Marrero et al. (2023) [71], Düzgün (2025) [72], and İzol et al. (2023) [73], suggesting a predominantly geogenic origin, with variability linked to soil properties. Toxic elements (Pb, Cd, As, Hg) remain low and within literature values, although slightly elevated Cd in polluted samples, as also reported by Babalau-Fuss et al. (2018) [69], may indicate localized contamination. Nickel and chromium fall within reported ranges (Bei et al.; İzol et al., 2023) [73], reflecting mixed natural and anthropogenic sources, while antimony and tin increase along the pollution gradient, supporting their association with anthropogenic inputs [74].

### 4.5. Implications for Food Safety and Human Exposure

**Figure 9:**
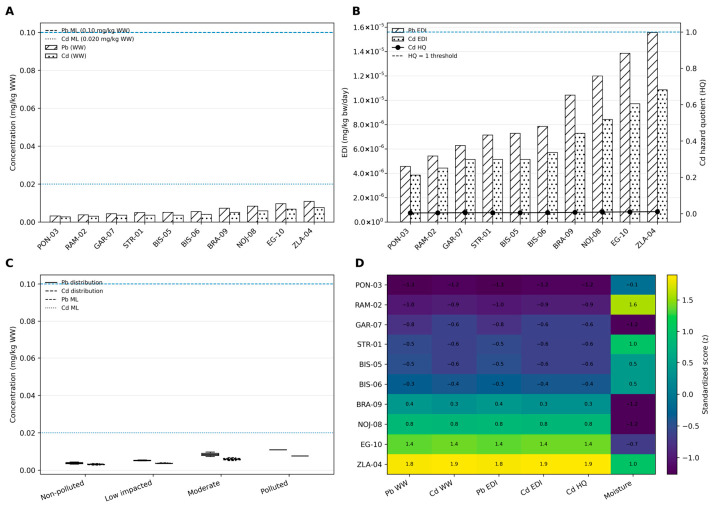
Integrated assessment of Pb and Cd concentrations, dietary exposure (EDI), and screening-level health risk indicators in *Prunus spinosa* fruits across a pollution gradient: (**A**) Pb and Cd concentrations in fruits compared with maximum limits (MLs); (**B**) Estimated daily intake (EDI) and hazard quotient (HQ) values for Cd exposure; (**C**) Distribution of Pb and Cd concentrations across pollution categories; (**D**) Heatmap visualization of standardized Pb/Cd-related exposure and risk indicators across sampling sites.

The food safety implications of *Prunus spinosa* fruits should be interpreted not only in relation to measured concentrations, but also in the context of dietary exposure and cumulative intake. As shown in Figure 9A, Pb and Cd concentrations across all sampling sites remained below the current European maximum levels for fresh fruits, suggesting low immediate health concern under the investigated conditions.

However, a clear spatial trend was observed, with increasing concentrations along the pollution gradient, particularly at sites affected by anthropogenic activities. This pattern may reflect differences in local environmental conditions and potential variations in metal availability. Previous studies have shown that soil-related processes, including acidification and changes in redox conditions, can influence the mobility of Fe/Mn-associated metals and the pool of plant-accessible metal species in contaminated environments. In addition, plant physiological responses to metal stress, including potential alterations in non-specific transport processes described under metal stress conditions, may further contribute to the accumulation of both essential and trace elements.

The implications of these concentration patterns become clearer when dietary exposure is considered. As illustrated in Figure 9B, the estimated daily intake (EDI) values for Pb and Cd increased progressively from non-polluted to polluted sites, reflecting the environmental contamination gradient and its potential influence on human intake. Despite this increase, the calculated screening-level risk indicators remained below levels of concern, indicating that consumption of these fruits is unlikely to pose a significant non-carcinogenic health risk under the current exposure assumptions. Although concentrations remained below regulatory thresholds, chronic low-dose exposure from repeated seasonal consumption should not be entirely disregarded.

Nevertheless, the concentration profiles across pollution categories (Figure 9C) highlight a consistent shift toward higher values in moderately and highly impacted environments. This observation reinforces the importance of environmental context, as even sub-threshold concentrations may contribute to long-term cumulative exposure when consumption is frequent, potentially affecting the overall nutritional and functional value of these fruits [75].

This trend is further supported by the z-score analysis (Figure 9D), which clearly differentiates sampling sites along the contamination gradient. Polluted locations (e.g., ZLA-04 and EG-10) were characterized by higher standardized values for Pb, Cd, and related exposure indicators (EDI and HQ), whereas non-polluted sites remained closer to background levels. This pattern may be consistent with soil–plant interactions affected by environmental contamination; whereby local contamination sources may influence both metal availability in soils and elemental uptake by plants. These findings indicate that harvesting location is an important determinant of the food safety quality of wild-harvested *Prunus spinosa* fruits.

### 4.6. Ecological and Geochemical Significance

The elemental profiles observed in *Prunus spinosa* fruits suggest the combined influence of lithogenic background, soil processes, and anthropogenic inputs. The increase in Al and Si in polluted sites may suggest enhanced deposition of soil-derived particles and atmospheric dust, indicating a stronger contribution of external inputs in impacted environments.

The contrasting trends of macroelements and trace elements point to changes in soil chemistry and plant uptake mechanisms. The decrease in K, Ca, and Mg, alongside the increase in Fe, Mn, Zn, and Cu, may be associated with differences in environmental conditions and potential variations in metal availability across the investigated sites. Previous studies have shown that altered soil pH and redox conditions can influence the mobility of Fe/Mn-associated metals in contaminated environments. In parallel, interactions between excess trace metals and essential nutrient uptake have been reported under contaminated environmental conditions and may contribute to altered membrane transport selectivity and nutrient balance.

Elemental ratios are also consistent with these processes. The decline in K/Na may be consistent with alterations in ionic balance and reduced selectivity between K^+^ and Na^+^ under stress conditions, while changes in Fe/Mn and Fe/Zn ratios may reflect differences in trace element mobility and uptake under varying environmental conditions.

Overall, these data indicate that *Prunus spinosa* occupies a functional interface between soil and atmosphere, integrating geogenic background signals with anthropogenic disturbance. This dual sensitivity strengthens its ecological relevance for landscape-scale biomonitoring.

### 4.7. Limitations of the Study

This study has several limitations. First, no direct soil analyses were performed, which limits the distinction between root uptake and atmospheric deposition pathways. In addition, fruit samples were washed prior to analysis to reduce superficial contamination and improve sample comparability; therefore, part of the loosely deposited particulate fraction may have been removed during sample preparation, potentially leading to underestimation of the total contribution of atmospheric particulate deposition for some lithogenic elements. Second, site classification along the environmental impact gradient was based on qualitative environmental descriptors rather than direct quantitative measurements of soil or atmospheric contamination. Although integrated contamination indices derived from fruit elemental composition were used as supportive tools, they do not replace direct environmental measurements. Third, sampling was conducted during a single season (2025), and therefore temporal variability was not assessed. Finally, only one species, *Prunus spinosa*, was investigated, and the findings should be interpreted at the species level. Because this study was observational and based on field sampling across naturally contrasting sites, the identified relationships should be interpreted as associations rather than direct causal effects. The multivariate analyses and integrated contamination indices should be interpreted as exploratory tools supporting comparative environmental assessment rather than direct quantitative measures of environmental contamination. Additional controlled studies integrating soil, air, and plant transfer pathways would be required to establish causality.

In addition, the food safety assessment was based exclusively on total elemental concentrations and did not include elemental speciation analysis, gastrointestinal bioaccessibility testing, or population-specific dietary exposure variability. Because elemental toxicity and human exposure may vary substantially depending on chemical form, bioavailability, and consumption patterns, the present food safety evaluation should be considered preliminary and screening-level in nature. Further studies integrating elemental speciation, bioaccessibility assessment, and refined dietary exposure modeling would be required for comprehensive toxicological characterization.

### 4.8. Future Research Directions

Building on the present findings, future studies should improve understanding of elemental accumulation in *Prunus spinosa* by integrating soil–plant analyses to assess bioavailability, transfer processes, and the effects of soil properties on metal mobility. Elemental speciation and bioaccessibility should also be examined, since total concentrations do not reflect the fraction relevant for human absorption. In vitro digestion or sequential extraction methods could improve dietary risk assessment. Long-term, multi-season monitoring is needed to distinguish stable spatial patterns from temporal variability. Expanding studies to other plant species would allow comparison of uptake strategies and improve biomonitoring relevance. Finally, multivariate approaches could strengthen source identification and predictive models, while further evaluation of *Prunus spinosa* as a bioindicator would support integrated environmental monitoring. 

## 5. Conclusions

The results indicate that the elemental composition of *Prunus spinosa* fruits is associated with environmental conditions and anthropogenic pressure gradients, with clear and statistically significant differences observed among site categories. Macroelements (K, Ca, Mg) generally decreased across sites classified within higher environmental pressure categories, while Na concentrations tended to increase, patterns that may be consistent with environmental stress and altered nutrient balance. In contrast, essential microelements such as Fe, Mn, Zn, and Cu showed higher concentrations in more impacted areas, whereas B and Mo displayed decreasing trends, potentially reflecting differences in elemental availability and uptake under varying environmental conditions. Geochemical trace elements (e.g., Al and Si) also increased progressively across the investigated site categories and may be associated with enhanced environmental exposure and particulate influence in impacted areas. Despite the accumulation of certain toxic elements, their concentrations remained below current regulatory limits, suggesting a generally low to moderate food safety risk depending on sample origin. Overall, *Prunus spinosa* fruits appear to have potential as bioindicators of environmental quality and anthropogenic influence under the investigated environmental conditions.

## Figures and Tables

**Table 1:** Summary of sampling design and distribution of biological samples across study areas and site categories.

Site Category (Environmental Pressure)	County	Number of Sites	Replicates per Site	Total Samples
Non-polluted	Alba	3	8	24
Low impacted	Alba	3	8	24
Low impacted	Bihor	1	8	8
Moderately impacted	Bihor	1	8	8
Moderately impacted	Cluj	1	8	8
Polluted	Alba	1	8	8
Total	—	10	8	80

**Table 2:** Concentration of macroelements in *Prunus spinosa* fruits across sampling sites (mg/kg DW).

Sample ID	Pollution Category	K (mg/kg)	Ca (mg/kg)	Mg (mg/kg)	P (mg/kg)	Na (mg/kg)	S (mg/kg)
RAM-02	Non-polluted	18,546 ± 315 ^ab^	1489 ± 62 ^b^	982 ± 41 ^a^	1422 ± 95 ^c^	497 ± 38 ^c^	5018.48 ± 61.20 ^a^
PON-03	Non-polluted	18,258 ± 210 ^b^	1562 ± 48 ^a^	912 ± 27 ^c^	1531 ± 72 ^b^	525 ± 44 ^c^	4983.29 ± 49.50 ^a^
GAR-07	Non-polluted	19,028 ± 405 ^a^	1530 ± 70 ^ab^	991 ± 55 ^a^	1473 ± 88 ^bc^	510 ± 36 ^c^	5026.77 ± 64.80 ^a^
STR-01	Low impacted	17,678 ± 265 ^c^	1430 ± 52 ^c^	943 ± 33 ^b^	1582 ± 110 ^a^	579 ± 60 ^b^	4969.15 ± 54.20 ^a^
BIS-05	Low impacted	18,088 ± 185 ^bc^	1512 ± 45 ^b^	892 ± 29 ^c^	1465 ± 68 ^c^	606 ± 55 ^b^	4988.74 ± 48.80 ^a^
BIS-06	Low impacted	17,362 ± 330 ^cd^	1466 ± 58 ^bc^	963 ± 38 ^ab^	1512 ± 82 ^b^	548 ± 41 ^bc^	4958.73 ± 57.60 ^a^
BRA-09	Low impacted	18,168 ± 275 ^bc^	1383 ± 49 ^cd^	910 ± 34 ^c^	1437 ± 76 ^c^	626 ± 63 ^b^	4947.03 ± 51.10 ^a^
NOJ-08	Moderately impacted	17,062 ± 420 ^d^	1454 ± 66 ^bc^	871 ± 31 ^d^	1629 ± 135 ^a^	698 ± 72 ^a^	4934.40 ± 62.50 ^a^
EG-10	Moderately impacted	17,840 ± 295 ^c^	1365 ± 57 ^d^	920 ± 36 ^bc^	1506 ± 90 ^b^	656 ± 68 ^ab^	4942.38 ± 55.40 ^a^
ZLA-04	Polluted	16,868 ± 360 ^d^	1420 ± 61 ^c^	880 ± 28 ^cd^	1411 ± 70 ^c^	721 ± 85 ^a^	4925.51 ± 60.00 ^a^

Lowercase superscript letters indicate significant differences between sampling sites according to Tukey’s post hoc test at *p* < 0.05.

**Table 3:** Concentration of essential microelements in *Prunus spinosa* fruits across sampling sites (mg/kg DW).

Sample ID	Pollution Category	Fe (mg/kg)	Mn (mg/kg)	Zn (mg/kg)	Cu (mg/kg)	B (mg/kg)	Mo (mg/kg)	Co (mg/kg)	Se (mg/kg)
RAM-02	Non-polluted	18.6 ± 2.1 ^c^	3.8 ± 0.5 ^c^	6.2 ± 0.7 ^c^	2.1 ± 0.3 ^c^	17.8 ± 1.4 ^a^	0.38 ± 0.04 ^a^	0.12 ± 0.02 ^c^	0.025 ± 0.003 ^c^
PON-03	Non-polluted	17.9 ± 1.8 ^c^	3.5 ± 0.4 ^c^	5.9 ± 0.6 ^c^	2.0 ± 0.2 ^c^	17.2 ± 1.3 ^a^	0.36 ± 0.04 ^a^	0.11 ± 0.02 ^c^	0.024 ± 0.003 ^c^
GAR-07	Non-polluted	20.3 ± 2.4 ^c^	4.2 ± 0.6 ^c^	6.8 ± 0.8 ^c^	2.3 ± 0.3 ^c^	18.5 ± 1.5 ^a^	0.40 ± 0.05 ^a^	0.13 ± 0.02 ^c^	0.026 ± 0.003 ^c^
STR-01	Low impacted	24.7 ± 2.8 ^b^	5.6 ± 0.7 ^b^	8.1 ± 0.9 ^b^	2.8 ± 0.3 ^b^	16.4 ± 1.3 ^b^	0.34 ± 0.03 ^b^	0.15 ± 0.02 ^b^	0.028 ± 0.003 ^b^
BIS-05	Low impacted	23.5 ± 2.6 ^b^	5.2 ± 0.6 ^b^	7.6 ± 0.8 ^b^	2.6 ± 0.3 ^b^	16.0 ± 1.2 ^b^	0.33 ± 0.03 ^b^	0.14 ± 0.02 ^b^	0.027 ± 0.003 ^b^
BIS-06	Low impacted	25.8 ± 3.0 ^b^	5.9 ± 0.7 ^b^	8.4 ± 0.9 ^b^	2.9 ± 0.3 ^b^	16.7 ± 1.3 ^b^	0.34 ± 0.03 ^b^	0.16 ± 0.02 ^b^	0.028 ± 0.003 ^b^
BRA-09	Low impacted	27.9 ± 3.2 ^b^	6.8 ± 0.8 ^b^	9.6 ± 1.1 ^b^	3.3 ± 0.4 ^b^	15.6 ± 1.3 ^b^	0.31 ± 0.03 ^b^	0.18 ± 0.03 ^b^	0.030 ± 0.004 ^b^
NOJ-08	Moderately impacted	31.4 ± 3.8 ^a^	7.9 ± 0.9 ^a^	11.2 ± 1.3 ^a^	3.8 ± 0.4 ^a^	14.8 ± 1.2 ^c^	0.29 ± 0.03 ^b^	0.20 ± 0.03 ^ab^	0.033 ± 0.004 ^a^
EG-10	Moderately impacted	33.8 ± 4.0 ^a^	8.6 ± 1.0 ^a^	12.4 ± 1.4 ^a^	4.2 ± 0.5 ^a^	14.2 ± 1.1 ^c^	0.27 ± 0.02 ^b^	0.22 ± 0.03 ^ab^	0.035 ± 0.004 ^a^
ZLA-04	Polluted	38.9 ± 4.5 ^a^	9.8 ± 1.1 ^a^	14.8 ± 1.6 ^a^	4.8 ± 0.5 ^a^	13.1 ± 1.0 ^c^	0.25 ± 0.02 ^b^	0.25 ± 0.04 ^a^	0.038 ± 0.005 ^a^

Lowercase superscript letters indicate significant differences between sampling sites according to Tukey’s post hoc test at *p* < 0.05.

**Table 4:** Geochemical trace element signature of *Prunus spinosa* fruits across sampling sites (mg/kg DW).

Sample ID	Pollution Category	Al (mg/kg)	Si (mg/kg)	Ba (mg/kg)	Sr (mg/kg)	Li (mg/kg)	Rb (mg/kg)	Cs (mg/kg)	V (mg/kg)	Ti (mg/kg)
RAM-02	Non-polluted	12.43 ± 1.47 ^c^	45.26 ± 4.83 ^c^	1.83 ± 0.21 ^c^	3.18 ± 0.37 ^c^	0.021 ± 0.004 ^c^	2.37 ± 0.29 ^c^	0.017 ± 0.003 ^c^	0.182 ± 0.021 ^c^	0.86 ± 0.11 ^c^
PON-03	Non-polluted	11.76 ± 1.34 ^c^	43.68 ± 4.57 ^c^	1.71 ± 0.19 ^c^	2.96 ± 0.33 ^c^	0.019 ± 0.003 ^c^	2.18 ± 0.27 ^c^	0.016 ± 0.002 ^c^	0.171 ± 0.019 ^c^	0.81 ± 0.10 ^c^
GAR-07	Non-polluted	13.28 ± 1.62 ^c^	47.61 ± 5.14 ^c^	1.94 ± 0.23 ^c^	3.36 ± 0.41 ^c^	0.022 ± 0.004 ^c^	2.52 ± 0.31 ^c^	0.018 ± 0.003 ^c^	0.194 ± 0.022 ^c^	0.89 ± 0.12 ^c^
STR-01	Low impacted	18.67 ± 2.14 ^b^	55.93 ± 6.07 ^b^	2.43 ± 0.28 ^b^	4.18 ± 0.52 ^b^	0.028 ± 0.005 ^b^	3.12 ± 0.41 ^b^	0.020 ± 0.003 ^b^	0.241 ± 0.028 ^b^	1.11 ± 0.13 ^b^
BIS-05	Low impacted	17.88 ± 2.03 ^b^	53.17 ± 5.63 ^b^	2.31 ± 0.27 ^b^	3.97 ± 0.48 ^b^	0.027 ± 0.004 ^b^	2.97 ± 0.39 ^b^	0.019 ± 0.003 ^b^	0.229 ± 0.027 ^b^	1.06 ± 0.12 ^b^
BIS-06	Low impacted	19.46 ± 2.21 ^b^	57.08 ± 6.25 ^b^	2.52 ± 0.29 ^b^	4.43 ± 0.54 ^b^	0.029 ± 0.005 ^b^	3.28 ± 0.42 ^b^	0.021 ± 0.003 ^b^	0.252 ± 0.029 ^b^	1.16 ± 0.14 ^b^
BRA-09	Low impacted	22.74 ± 2.63 ^b^	62.31 ± 6.84 ^b^	2.88 ± 0.32 ^b^	5.08 ± 0.61 ^b^	0.033 ± 0.006 ^b^	3.79 ± 0.47 ^b^	0.023 ± 0.004 ^b^	0.287 ± 0.031 ^b^	1.29 ± 0.16 ^b^
NOJ-08	Moderately impacted	27.63 ± 3.19 ^a^	71.42 ± 7.86 ^a^	3.58 ± 0.41 ^a^	6.27 ± 0.72 ^a^	0.041 ± 0.007 ^a^	4.63 ± 0.58 ^a^	0.026 ± 0.004 ^a^	0.362 ± 0.038 ^a^	1.53 ± 0.18 ^a^
EG-10	Moderately impacted	30.84 ± 3.57 ^a^	78.15 ± 8.47 ^a^	4.12 ± 0.49 ^a^	7.08 ± 0.83 ^a^	0.046 ± 0.008 ^a^	5.18 ± 0.64 ^a^	0.029 ± 0.004 ^a^	0.403 ± 0.044 ^a^	1.71 ± 0.20 ^a^
ZLA-04	Polluted	36.58 ± 4.18 ^a^	88.73 ± 9.62 ^a^	4.83 ± 0.57 ^a^	8.36 ± 0.94 ^a^	0.053 ± 0.009 ^a^	6.07 ± 0.72 ^a^	0.034 ± 0.005 ^a^	0.482 ± 0.051 ^a^	1.94 ± 0.23 ^a^

Lowercase superscript letters indicate significant differences between sampling sites according to Tukey’s post hoc test at *p* < 0.05.

**Table 5:** Toxic elements in *Prunus spinosa* fruits (mg/kg DW and WW).

Sample ID	Pollution Category	Pb (mg/kg) DW	Pb (mg/kg) WW	Cd (mg/kg) DW	Cd (mg/kg) WW	As (mg/kg) DW	Hg (mg/kg) DW
Maximum Allowed Limit		-	0.10 ML (mg/kg WW)	-	0.020 ML (mg/kg WW)	-	-
RAM-02	Non-polluted	0.021 ± 0.004 ^c^	0.0038 ± 0.0007 ^c^	0.017 ± 0.003 ^c^	0.0031 ± 0.0005 ^c^	0.186 ± 0.009 ^c^	0.019 ± 0.003 ^c^
PON-03	Non-polluted	0.019 ± 0.003 ^c^	0.0032 ± 0.0005 ^c^	0.016 ± 0.002 ^c^	0.0027 ± 0.0003 ^c^	0.171 ± 0.019 ^c^	0.018 ± 0.003 ^c^
GAR-07	Non-polluted	0.022 ± 0.004 ^c^	0.0044 ± 0.0008 ^c^	0.018 ± 0.003 ^c^	0.0036 ± 0.0006 ^c^	0.194 ± 0.022 ^c^	0.020 ± 0.003 ^c^
STR-01	Low impacted	0.028 ± 0.005 ^b^	0.0050 ± 0.0009 ^b^	0.020 ± 0.003 ^b^	0.0036 ± 0.0005 ^b^	0.241 ± 0.028 ^b^	0.023 ± 0.004 ^b^
BIS-05	Low impacted	0.027 ± 0.004 ^b^	0.0051 ± 0.0008 ^b^	0.019 ± 0.003 ^b^	0.0036 ± 0.0006 ^b^	0.229 ± 0.027 ^b^	0.021 ± 0.003 ^b^
BIS-06	Low impacted	0.029 ± 0.005 ^b^	0.0055 ± 0.0009 ^b^	0.021 ± 0.003 ^b^	0.0040 ± 0.0006 ^b^	0.252 ± 0.029 ^b^	0.024 ± 0.004 ^b^
BRA-09	Low impacted	0.033 ± 0.006 ^b^	0.0073 ± 0.0013 ^b^	0.023 ± 0.004 ^b^	0.0051 ± 0.0009 ^b^	0.287 ± 0.031 ^b^	0.026 ± 0.004 ^b^
NOJ-08	Moderately impacted	0.038 ± 0.007 ^ab^	0.0084 ± 0.0015 ^ab^	0.027 ± 0.005 ^ab^	0.0059 ± 0.0011 ^ab^	0.331 ± 0.036 ^ab^	0.030 ± 0.005 ^ab^
EG-10	Moderately impacted	0.044 ± 0.008 ^a^	0.0097 ± 0.0018 ^a^	0.031 ± 0.005 ^a^	0.0068 ± 0.0011 ^a^	0.368 ± 0.040 ^a^	0.034 ± 0.006 ^a^
ZLA-04	Polluted	0.052 ± 0.009 ^a^	0.0109 ± 0.0019 ^a^	0.036 ± 0.006 ^a^	0.0076 ± 0.0013 ^a^	0.421 ± 0.045 ^a^	0.039 ± 0.007 ^a^

Lowercase superscript letters indicate significant differences between sampling sites according to Tukey’s post hoc test at *p* < 0.05.

**Table 6:** Potentially toxic elements (Ni and Cr) in samples across different pollution categories (mg/kg DW).

Sample ID	Pollution Category	Ni (mg/kg) DW	Cr (mg/kg) DW
RAM-02	Non-polluted	1.83 ± 0.21 ^c^	3.18 ± 0.37 ^c^
PON-03	Non-polluted	1.71 ± 0.19 ^c^	2.96 ± 0.33 ^c^
GAR-07	Non-polluted	1.94 ± 0.23 ^c^	3.36 ± 0.41 ^c^
STR-01	Low impacted	2.43 ± 0.28 ^b^	4.18 ± 0.52 ^b^
BIS-05	Low impacted	2.31 ± 0.27 ^b^	3.97 ± 0.48 ^b^
BIS-06	Low impacted	2.52 ± 0.29 ^b^	4.43 ± 0.54 ^b^
BRA-09	Low impacted	2.88 ± 0.32 ^b^	5.08 ± 0.61 ^b^
NOJ-08	Moderately impacted	3.36 ± 0.38 ^ab^	5.94 ± 0.72 ^ab^
EG-10	Moderately impacted	3.82 ± 0.44 ^a^	6.71 ± 0.81 ^a^
ZLA-04	Polluted	4.47 ± 0.52 ^a^	7.88 ± 0.94 ^a^

Lowercase superscript letters indicate significant differences between sampling sites according to Tukey’s post hoc test at *p* < 0.05.

**Table 7:** Additional contaminant-related elements (Sb and Sn) in samples across different pollution categories (mg/kg DW).

Sample ID	Pollution Category	Sb (mg/kg) DW	Sb (mg/kg WW)	Sn (mg/kg) DW
RAM-02	Non-polluted	2.34 ± 045 ^c^	0.4029 ± 0.0493 ^c^	12.08 ± 0.95 ^c^
PON-03	Non-polluted	2.18 ± 0.27 ^c^	0.4360 ± 0.0540 ^c^	11.76 ± 1.34 ^c^
GAR-07	Non-polluted	2.52 ± 0.31 ^c^	0.5544 ± 0.0682 ^b^	13.28 ± 1.62 ^c^
STR-01	Low impacted	3.12 ± 0.41 ^b^	0.5616 ± 0.0738 ^b^	18.67 ± 2.14 ^b^
BIS-05	Low impacted	2.97 ± 0.39 ^b^	0.5643 ± 0.0741 ^b^	17.88 ± 2.03 ^b^
BIS-06	Low impacted	3.28 ± 0.42 ^b^	0.6232 ± 0.0798 ^b^	19.46 ± 2.21 ^b^
BRA-09	Low impacted	3.79 ± 0.47 ^b^	0.8338 ± 0.1034 ^ab^	22.74 ± 2.63 ^b^
NOJ-08	Moderately impacted	4.46 ± 0.55 ^ab^	0.9812 ± 0.1210 ^a^	26.91 ± 3.08 ^ab^
EG-10	Moderately impacted	5.02 ± 0.61 ^a^	1.0542 ± 0.1281 ^a^	30.84 ± 3.57 ^a^
ZLA-04	Polluted	5.88 ± 0.72 ^a^	1.0584 ± 0.1296 ^a^	36.58 ± 4.18 ^a^

Note: No maximum regulatory limits are established for Sb in fresh fruits under current European Union legislation (Commission Regulation (EU) 2023/915) or Codex Alimentarius standards (CXS 193-1995). For Sn, a maximum level of 200 mg/kg (wet weight) is established only for processed products such as canned foods and is therefore not applicable to the present dataset. Lowercase superscript letters indicate significant differences between sampling sites according to Tukey’s post hoc test at *p* < 0.05.

**Table 8:** Principal component analysis (PCA) loadings and ecological interpretation of elements along the pollution gradient.

Element	PC1 Loading	PC2 Loading	Direction (PC1)	Trend Across Site Categories	Element Group	Observed Pattern Across Site Categories
K	−0.250	0.293	Negative	↓ decreasing	Macroelement	Generally higher in non-polluted site categories and lower in more impacted categories
Ca	−0.234	−0.543	Negative	↓ decreasing	Macroelement	Lower concentrations observed in more impacted site categories
Mg	−0.221	0.682	Negative	↓ decreasing	Macroelement	Lower values in more impacted site categories
Na	0.299	−0.197	Positive	↑ increasing	Macroelement	Tends to increase across the site classification gradient
Fe	0.306	0.111	Positive	↑ increasing	Essential microelement	Higher concentrations observed in more impacted site categories
Mn	0.307	0.121	Positive	↑ increasing	Essential microelement	Higher concentrations in more impacted site categories
Zn	0.304	0.114	Positive	↑ increasing	Essential microelement	Higher concentrations in site categories classified as more impacted
Cu	0.306	0.129	Positive	↑ increasing	Essential microelement	Shows increasing concentrations across the site classification gradient
B	−0.305	0.121	Negative	↓ decreasing	Essential element	Decreases across the site classification gradient
Mo	−0.305	0.095	Negative	↓ decreasing	Essential trace element	Lower concentrations in more impacted site categories
Al	0.308	0.084	Positive	↑ increasing	Lithogenic element	Higher concentrations in more impacted site categories
Si	0.300	0.167	Positive	↑ increasing	Lithogenic element	Higher concentrations observed in more impacted site categories

↑ indicates increasing trend across site categories; ↓ indicates decreasing trend across site categories.

## Data Availability

The original contributions presented in this study are included in the article/Supplementary Materials. Further inquiries can be directed to the corresponding authors.

## Supplementary Materials

The following supporting information can be downloaded at: https://www.mdpi.com/article/10.3390/foods15101726/s1. Table S1: Sampling sites and characterization of mature *Prunus spinosa* L. fruit samples collected across the investigated environmental gradient; Table S2: Classification of sampling sites according to pollution risk and environmental pressure; Table S3: Distribution of biological samples across sampling sites, counties, and pollution categories; Table S4: Sample preparation procedure (washing, drying, grinding, and storage); Table S5: Microwave-assisted acid digestion conditions for sample preparation; Table S6: ICP–MS instrumental operating conditions (Thermo Scientific iCAP Q, Bremen, Germany); Table S7: Quality assurance and quality control (QA/QC) protocol for ICP-MS analysis; Table S8: Evaluation of analytical accuracy and precision for multi-element determination by ICP-MS based on certified reference material and quality assurance/quality control (QA/QC) criteria; Table S9: Analytical performance characteristics for multi-element determination by ICP-MS; Table S10: Overview of Regulatory Limits, Analytical Comparability, and Interpretation of Trace Elements in Fresh Fruits; Table S11. Integrated macroelement profile, variability, and ecological indicators in *Prunus spinosa* fruits across pollution gradients; Table S12: Integrated trace element profile, variability, and ecological indicators in *Prunus spinosa* fruits (mg/kg DW) across pollution gradients; Table S13: Integrated geochemical trace element profile, variability, and environmental indicators in *Prunus spinosa* fruits across pollution gradients (mg/kg DW); Table S14: Conversion of selected elemental concentrations from dry weight (DW) to wet weight (WW) in *Prunus spinosa* fruits; Table S15: Integrated toxic and contaminant element profile, variability, and environmental indicators in *Prunus spinosa* fruits across pollution gradients (mg/kg DW).

## Abbreviations

The following abbreviations are used in this manuscript:

ICP-MS	Inductively Coupled Plasma Mass Spectrometry
QA/QC	Quality Assurance/Quality Control
CRM	Certified Reference Material
NIST	National Institute of Standards and Technology
DW	Dry Weight
WW	Wet Weight
LOD	Limit of Detection
LOQ	Limit of Quantification
BEC	Background-Equivalent Concentration
RSD	Relative Standard Deviation
ANOVA	Analysis of Variance
DEM	Digital Elevation Model
WGS84	World Geodetic System 1984
PTFE	Polytetrafluoroethylene
HDPE	High-Density Polyethylene
EU	European Union
FAO	Food and Agriculture Organization
WHO	World Health Organization
ML	Maximum Level
Ionome	Total elemental composition of a biological organism or tissue
