# Supplementary material for: Multi-Element Composition of Wild Prunus spinosa Fruits Across Contrasting Environments: Implications for Food Safety and Quality
Source: Foods. 2026 May 14;15(10):1726. doi: 10.3390/foods15101726 (PMC13206278; doi:10.3390/foods15101726)
Supplement: Supplementary file 1 [file foods-15-01726-s001.zip › foods-4307443-supplementary.pdf]

Table S1. Sampling sites and characterization of mature Prunus spinosa L. fruit samples collected across the investigated environmental gradient.

No.	Sample ID	County	Administrative unit	Locality	Latitude (N)	Longitude (E)	Altitude (m a.s.l.)	Habitat type	Land use	Anthropogenic pressure	Ecological remarks
1	STR-01	Alba	Stremț Commune	Stremț	46°15'28''	23°35'53''	~350	Rural	Agricultural / mixed	Low	Spontaneous shrubs at field margins
2	RAM-02	Alba	Râmeț Commune	Cotorăști	46°19'51''	23°32'45''	~500	Mountain	Grassland / forest	Very low	Calcareous (karst) substrate
3	PON-03	Alba	Ponor Commune	Ponor	46°19'34''	23°22'37''	~550	Mountain	Grassland	Low	Karstic relief, well-drained soils
4	ZLA-04	Alba	Zlatna Town	Valea Mică	46°41'45''	23°16'45''	~450	Hilly	Mixed	High	Mining-impacted area (metal contamination risk)
5	BIS-05	Alba	Bistra Commune	Crețești	46°21'28''	23°06'21''	~600	Mountain	Forest	Low	Variable geological substrate
6	BIS-06	Alba	Bistra Commune	Aronești	46°26'17''	23°08'12''	~650	Mountain	Forest	Low	Relatively stable ecosystem
7	GAR-07	Alba	Gârda de Sus Commune	Dealul Sturului	46°30'22''	22°49'02''	~900	Mountain	Grassland	Very low	Apuseni Mts., limestone influence
8	NOJ-08	Bihor	Nojorid Commune	Leș	46°57'57''	21°50'09''	~150	Plain	Intensive agriculture	Moderate	Possible fertilizer influence

9	BRA-09	Bihor	Bratca Commune	Beznea	46°56'38.8"	22°42'38.6"	~400	Mountain	Forest	Low	Geological transition zone
10	EG-10	Cluj	Negreni Commune	Bucea	46°56′00.83″	22°42′38.6″	~420*	Mountain	Mixed / roadside	Moderate–high	Proximity to major traffic corridor

Table S2. Classification of sampling sites according to pollution risk and environmental pressure

No.	Sample ID	Locality	Pollution category	Main pollution source	Agricultural intensity	Traffic influence	Environmental risk level	Justification
1	STR-01	Stremț	Low impacted	Diffuse (background)	Moderate	Low	Low	Rural area, limited anthropogenic influence
2	RAM-02	Cotorăști	Non-polluted	Natural background	Low	Very low	Very low	Remote karst area, minimal human activity
3	PON-03	Ponor	Non-polluted	Natural background	Low	Very low	Very low	Mountain grassland, low disturbance
4	ZLA-04	Valea Mică	Polluted	Mining activities (historical)	Low	Moderate	High	Known mining area, potential heavy metal contamination
5	BIS-05	Crețești	Low impacted	Geological background	Low	Low	Low	Forested area, minimal external inputs
6	BIS-06	Aronești	Low impacted	Geological background	Low	Low	Low	Stable mountain ecosystem
7	GAR-07	Dealul Sturului	Non-polluted	Natural background	Very low	Very low	Very low	High-altitude area, isolated
8	NOJ-08	Leș	Moderately impacted	Agriculture (fertilizers, pesticides)	High	Low	Moderate	Intensive agricultural practices
9	BRA-09	Beznea	Low impacted	Mixed (natural + minor anthropogenic)	Low	Moderate	Low–moderate	Transitional zone, some human influence
10	NEG-10	Bucea	Moderately impacted	Road traffic + mixed sources	Moderate	High	Moderate–high	Proximity to major traffic corridor

Table S3. Distribution of biological samples across sampling sites, counties, and pollution categories

No.	Sample ID	County	Locality	Pollution category	Replicates per site	Total samples	Environmental pressure	Main influencing factor
1	STR-01	Alba	Stremț	Low impacted	8	8	Low	Rural background
2	RAM-02	Alba	Cotorăști	Non-polluted	8	8	Very low	Natural karst system
3	PON-03	Alba	Ponor	Non-polluted	8	8	Very low	Mountain grassland
4	ZLA-04	Alba	Valea Mică	Polluted	8	8	High	Mining activities
5	BIS-05	Alba	Crețești	Low impacted	8	8	Low	Forest ecosystem
6	BIS-06	Alba	Aronești	Low impacted	8	8	Low	Stable mountain system
7	GAR-07	Alba	Gârda de Sus	Non-polluted	8	8	Very low	High-altitude isolated area
8	NOJ-08	Bihor	Leș	Moderately impacted	8	8	Moderate	Intensive agriculture
9	BRA-09	Bihor	Beznea	Low impacted	8	8	Low–moderate	Mixed natural-anthropogenic
10	EG-10	Cluj	Bucea	Moderately impacted	8	8	Moderate–high	Traffic emissions

Table S4. Sample preparation procedure (washing, drying, grinding, and storage)

Stage	Step	Procedure	Conditions / Parameters
Washing	1	Rinsing with distilled water	2–3 times
	2	Gentle agitation	~1–2 min
	3	Final rinse	Distilled water, 1 time
	4	Surface drying	Filter paper, room temperature
Drying	5	Oven drying	40–60 °C
	6	Drying duration	24–72 h (until constant weight)
	7	Cooling	Desiccator
Grinding / Homogenization	8	Pre-cutting (if necessary)	Stainless steel tools
	9	Grinding	Laboratory mill
	10	Sieving (optional)	~0.5 mm mesh
	11	Homogenization	Manual mixing
	12	Cleaning equipment	Ethanol / distilled water
Storage	13	Storage containers	Polyethylene / glass
	14	Storage temperature	Room temperature or 4 °C
	15	Light conditions	Protected from light
	16	Humidity control	Dry conditions
	17	Labeling	Sample ID + date

Table S5. Microwave-assisted acid digestion conditions for sample preparation

Category	Parameter	Specification / Condition
Instrumentation	Digestion system	START D Microwave Digestion System
	Digestion mode	Closed-vessel microwave digestion
	Vessel type	PTFE high-pressure vessels (50–100 mL)
Sample preparation	Sample mass	0.300–0.500 g
	Sample condition	Dried and homogenized
Reagents	HNO_3	6 mL (65%, analytical grade)
	H_2O_2	2 mL (30%, analytical grade)
	Sample/acid ratio	~1:16 (w/v)
Pre-digestion	Reaction step	10 min at room temperature
Microwave program	Heating profile	Controlled ramp: 120 °C (5 min), hold 5 min; 180 °C (10 min), hold 15 min
Operating conditions	Max temperature	180 °C
	Max pressure	≤ 30 bar
	Power	Up to 1000 W (automatic)
	Temperature control	Internal sensor / automatic regulation
Post-digestion	Cooling	To <50 °C before opening
Final preparation	Dilution	To 25–50 mL with ultrapure water
Quality control	Blanks	Reagent blanks included
	Replicates	n = 3

Table S6. ICP–MS instrumental operating conditions (Thermo Scientific iCAP Q, Bremen, Germany)

Category	Parameter	Specification / Setting	Analytical significance
Instrument	ICP-MS system	Thermo Scientific iCAP Q (quadrupole ICP-MS)	High-sensitivity multi-element detection
Plasma conditions	RF power	1550 W	Stable plasma generation
	Plasma gas flow (Ar)	14.0 L/min	Plasma maintenance
	Auxiliary gas flow	0.8 L/min	Plasma positioning
	Nebulizer gas flow	0.95–1.05 L/min (daily optimized)	Aerosol transport efficiency
Sample introduction	Nebulizer type	Concentric nebulizer (glass)	Fine aerosol generation
	Spray chamber	Quartz cyclonic spray chamber	Droplet size control
Ion optics	Sample uptake rate	~1.0 mL/min	Stable sample delivery
	Interface cones	Ni sampler and skimmer cones	Ion extraction efficiency
	Ion focusing	Electrostatic lenses	Signal optimization
Collision/reaction cell	Mode	Kinetic Energy Discrimination (KED)	Interference removal
	Collision gas	Helium (He)	Reduction of polyatomic interferences
	He gas flow	~4.5 mL/min	Optimization of collision efficiency
Acquisition parameters	Scan mode	Peak hopping	Multi-element acquisition
	Dwell time	10–50 ms per isotope	Signal integration
	Sweeps per reading	≥ 20	Signal stability
	Replicates per sample	3	Precision improvement
Detection system	Detector type	Dual mode (pulse and analog)	Wide dynamic range
Isotope selection	Analytical isotopes	Element-specific (interference-free isotopes selected)	Accuracy
Internal standardization	Internal standards	Rh (^{103}Rh), In (^{115}In), Bi (^{209}Bi)	Drift and matrix correction
Interference control	Polyatomic correction	He-KED mode	Reduction of Ar-based interferences
Calibration	Calibration type	External multi-element calibration	Quantitative analysis

	Calibration verification	Check standards every 10–15 samples	Instrument stability
Rinse procedure	Wash solution	2–5% HNO_3	Prevention of carry-over
Detection performance	Sensitivity	Optimized daily using tuning solution	Maximum signal intensity
	Background equivalent concentration (BEC)	Monitored per element	Noise control
Quality control	Tuning solution	Multi-element tuning solution	Daily performance validation

Table S7. Quality assurance and quality control (QA/QC) protocol for ICP-MS analysis

Category	Parameter	Specification / Condition	Acceptance criteria	Analytical purpose
Sample analysis	Replicates (biological + analytical)	n = 3 (independent digestion and measurement)	RSD ≤ 5%	Assessment of repeatability and analytical precision
Blanks	Reagent blanks	Included in each digestion batch (same reagents and procedure)	Signal < LOD	Detection of contamination and background correction
Method blanks	Procedural blanks	Full digestion without sample	No significant signal	Verification of digestion purity
Calibration	Calibration strategy	External multi-element calibration	Linear response	Quantitative determination
	Calibration points	Minimum 5 concentration levels	R^2 typically ≥ 0.998–0.999, depending on analyte concentration range and signal intensity	Linearity validation
	Calibration matrix	Acidified (HNO_3, matched to samples)	Matrix consistency	Minimization of matrix effects
Internal standards	Internal standard elements	Rh, In, Bi (multi-point correction)	Stable signal (±5%)	Correction of drift and matrix suppression
Instrument performance	Daily tuning	Sensitivity, oxide ratio (CeO^+/Ce^+), doubly charged ions (Ce^{2+}/Ce^+)	$CeO^+/Ce^+ < 2\%$; $Ce^{2+}/Ce^+ < 3\%$	Optimization of plasma conditions

Accuracy	Certified reference material (CRM)	Multiple certified reference materials (CRMs), including botanical, geological, and mixed-environment matrices	Recovery: 90–110%	Validation of method accuracy
Precision	Repeatability (intra-day)	Triplicate measurements	RSD ≤ 5%	Measurement consistency
	Reproducibility (inter-batch)	Independent digestion batches	RSD ≤ 10%	Method robustness
Recovery test	Spike recovery	Addition of known standard concentration	90–110%	Matrix effect evaluation
Detection limits	LOD	3σ of blank signal	Element-specific	Sensitivity determination
	LOQ	10σ of blank signal	Element-specific	Quantification threshold
Instrument stability	Drift control	Periodic recalibration (every 10–15 samples)	±5% deviation	Signal stability
Carry-over	Wash procedure	Acid wash (HNO_3 2–5%) between samples	No memory effect	Prevention of cross-contamination
Data validation	Outlier control	Grubbs or Dixon test (if applicable)	$p < 0.05$	Statistical reliability
Units and reporting	Expression of results	mg/kg dry weight (DW)	Consistent basis	Data comparability

Table S8. Evaluation of analytical accuracy and precision for multi-element determination by ICP-MS based on certified reference material and quality assurance/quality control (QA/QC) criteria

Element	Category	CRM (MRC source)	Certified value (mg/kg)	Measured value (mg/kg)	Recovery (%)	RSD (%)
			Macroelements			
K	Macroelement	NIST SRM 1573a	26760 ± 480	26185	97.9	2.4
Ca	Macroelement	NIST SRM 1573a	50450 ± 550	49290	97.7	2.1
Mg	Macroelement	NIST SRM 1515	2710 ± 80	2684	99.0	2.7
P	Macroelement	NIST SRM 1573a	2161 ± 28	2118	98.0	2.8
Na	Macroelement	NIST SRM 1573a	136.1 ± 3.7	132.8	97.6	3.4
S	Macroelement	NIST SRM 1547	9600 ± 300	9485	98.8	3.1
			Microelements			
Fe	Microelement	NIST SRM 1573a	367.5 ± 4.3	359.6	97.9	2.7
Mn	Microelement	NIST SRM 1573a	246.3 ± 7.1	241.1	97.9	2.9
Zn	Microelement	NIST SRM 1573a	30.94 ± 0.55	30.21	97.6	3.1
Cu	Microelement	NIST SRM 1573a	4.70 ± 0.14	4.58	97.4	3.5
B	Microelement	NIST SRM 1573a	33.13 ± 0.42	32.48	98.0	2.6
Mo	Microelement	NIST SRM 1573a	0.46 ± 0.05	0.45	97.8	4.0
Co	Microelement	NIST SRM 1573a	0.5773 ± 0.0071	0.563	97.5	3.8
Se	Microelement	NIST SRM 1573a	0.0543 ± 0.0020	0.0529	97.4	4.2
			Heavy metals / toxic elements			
Pb	Toxic element	NIST SRM 1515	0.47 ± 0.02	0.45	95.7	4.3
Cd	Toxic element	NIST SRM 1573a	1.517 ± 0.027	1.486	97.9	3.0
As	Toxic element	NIST SRM 1573a	0.1126 ± 0.0024	0.1099	97.6	4.1
Hg	Toxic element	NIST SRM 1573a	0.0341 ± 0.0015	0.0332	97.4	4.5
Ni	Toxic element	NIST SRM 1573a	1.582 ± 0.041	1.549	97.9	3.3
Cr	Toxic element	NIST SRM 1573a	1.988 ± 0.034	1.944	97.8	3.0
Sb	Toxic element	BCR-129	0.065 ± 0.005	0.061	93.8	4.4
Sn	Toxic element	BCR-129	0.20 ± 0.02	0.19	95.0	4.1
			Geochemical / lithogenic / trace elements			
Al	Lithogenic	NIST SRM 1573a	598.4 ± 7.1	586.7	98.0	2.5
Si	Lithogenic	NIST SRM 2711a	1500 ± 50	1450	96.7	3.7

Ba	Trace element	NIST SRM 1515	63 ± 3	62.0	98.4	3.3
Sr	Trace element	NIST SRM 1515	85 ± 4	83.5	98.2	3.1
Li	Trace element	GBW 07603	0.30 ± 0.03	0.28	93.3	4.2
Rb	Trace element	NIST SRM 1573a	14.83 ± 0.31	14.49	97.7	3.2
Cs	Trace element	GBW 07603	0.055 ± 0.005	0.052	94.5	4.4
V	Trace element	NIST SRM 1573a	0.835 ± 0.034	0.815	97.6	4.0

Note: CRM, certified reference material; RSD, relative standard deviation. Analytical accuracy was evaluated using certified reference materials (NIST SRM 1573a, 1515, 1547, 2711a; BCR-129; GBW 07603). Recovery (%) was calculated as the ratio of measured to certified values, and precision was expressed as RSD (%).

Table S9. Analytical performance characteristics for multi-element determination by ICP-MS.

Element	Linear range (μg kg⁻¹)	R^2	LOD (μg kg⁻¹)	LOQ (μg kg⁻¹)	BEC (μg kg⁻¹)
K	4825–51870	0.9986	873.4	2647.0	342.8
Ca	2175–60120	0.9988	512.6	1553.3	191.4
Mg	1195–19740	0.9991	268.9	814.7	96.2
P	2460–29780	0.9984	734.2	2224.8	289.5
Na	3155–40120	0.9983	826.7	2505.2	305.1
S	5330–78640	0.9981	1187.3	3598.9	468.6
Fe	14.7–4985	0.9996	5.37	16.28	2.63
Mn	2.8–1975	0.9997	1.08	3.27	0.58
Zn	0.98–972	0.9997	0.52	1.57	0.24
Cu	1.15–486	0.9996	0.31	0.94	0.13
B	5.9–1960	0.9993	2.14	6.49	0.93
Mo	0.47–193	0.9998	0.11	0.34	0.06
Co	0.28–96	0.9998	0.054	0.164	0.027
Se	0.58–188	0.9997	0.12	0.36	0.06
Pb	0.47–512	0.9998	0.21	0.64	0.09
Cd	0.11–96	0.9999	0.053	0.161	0.026
As	0.63–182	0.9998	0.11	0.34	0.05
Hg	0.09–47	0.9997	0.052	0.158	0.026
Ni	0.95–488	0.9997	0.32	0.98	0.12
Cr	1.22–452	0.9996	0.31	0.95	0.11
Sb	0.12–94	0.9997	0.054	0.163	0.027
Al	62–9830	0.9994	14.6	44.2	6.3
Si	515–49620	0.9982	82.4	249.7	36.8
Ba	0.97–965	0.9997	0.53	1.61	0.25
Sr	1.18–942	0.9997	0.51	1.55	0.24
Li	0.21–188	0.9998	0.11	0.33	0.05
Rb	0.52–476	0.9997	0.21	0.64	0.09
Cs	0.11–93	0.9998	0.052	0.158	0.026
V	0.57–186	0.9997	0.12	0.35	0.05
Ti	2.15–972	0.9994	1.08	3.27	0.52

Abbreviations: LOD = limit of detection; LOQ = limit of quantification; BEC = background equivalent concentration.

Table S10. Overview of Regulatory Limits, Analytical Comparability, and Interpretation of Trace Elements in Fresh Fruits

Element	EU regulatory maximum level (mg/kg WW)	Codex Alimentarius status (CXS 193-1995)	Applicable food category	Analytical comparability with dataset	Interpretative framework
Pb (Lead)	0.10	Established ML: 0.10 mg/kg for fruits (including stone fruits)	Fresh fruits (raw commodities)	Comparable following harmonization of expression basis (DW/WW)	Primary toxicological indicator; key element for regulatory compliance assessment
Cd (Cadmium)	0.020	No specific ML identified for fresh fruits	Fresh fruits (including stone fruits)	Comparable following harmonization of expression basis (DW/WW)	Critical contaminant under EU framework; interpreted within food safety context
As (Arsenic)	Not specified for fresh fruits	No ML for raw fruits (limits exist for derived products)	Processed matrices (e.g., juices)	Not directly comparable	Environmental contaminant; interpreted within geochemical and exposure context
Hg (Mercury)	Not specified for fresh fruits	No ML for raw fruits	Primarily fishery products	Not directly comparable	Contextual contaminant; low relevance for plant-derived matrices
Ni (Nickel)	Not regulated	No Codex ML for fruits	—	Not directly comparable	Evaluated as ecotoxicological and soil-derived trace element
Cr (Chromium)	Not regulated	No Codex ML for fruits	—	Not directly comparable	Interpreted as lithogenic and anthropogenic input indicator
Sb (Antimony)	Not regulated	No Codex ML for fruits	—	Not directly comparable	Trace-level contaminant; indicative of anthropogenic influence
Sn (Tin, inorganic)	200 (canned foods) / 100 (beverages)	ML applicable to processed products only	Canned foods and beverages	Not comparable	Not relevant for fresh fruit assessment; processing-related contaminant
Cu (Copper)	Not regulated as contaminant	No Codex ML for fruits	Essential micronutrient	Not applicable	Interpreted within nutritional and physiological framework
Zn (Zinc)	Not regulated as contaminant	No Codex ML for fruits	Essential micronutrient	Not applicable	Nutritional and enzymatic cofactor; ecological indicator
Fe (Iron)	Not regulated as contaminant	No Codex ML for fruits	Essential micronutrient	Not applicable	Nutritional relevance; redox-active element reflecting soil composition

Mn (Manganese)	Not regulated as contaminant	No Codex ML for fruits	Essential micronutrient	Not applicable	Plant metabolic element; indicator of pedological conditions
B (Boron)	Not regulated	No Codex ML	Essential trace element	Not applicable	Strongly controlled by soil geochemistry; sensitive ecological marker
Mo (Molybdenum)	Not regulated	No Codex ML	Essential trace element	Not applicable	Enzymatic cofactor; typically present at ultra-trace levels
Co (Cobalt)	Not regulated	No Codex ML	Trace element	Not applicable	Geochemical background element; minor biological relevance
Se (Selenium)	Not regulated for fruits	No Codex ML	Trace element	Not applicable	Nutritional trace element; variability linked to soil Se availability
Al (Aluminium)	Not regulated for fruits	No Codex ML	Lithogenic element	Not applicable	Strong indicator of soil-derived particulate input
Si (Silicon)	Not regulated	No Codex ML	Lithogenic element	Not applicable	Reflects mineral substrate and dust deposition
Ba (Barium)	Not regulated	No Codex ML	Trace element	Not applicable	Geological tracer; low biological relevance
Sr (Strontium)	Not regulated	No Codex ML	Trace element	Not applicable	Proxy for Ca-related geochemical pathways
Li (Lithium)	Not regulated	No Codex ML	Trace element	Not applicable	Environmental trace element; low concentrations in plant tissues
Rb (Rubidium)	Not regulated	No Codex ML	Trace element	Not applicable	Alkali metal analogue; reflects soil mineralogy
Cs (Cesium)	Not regulated	No Codex ML	Trace element	Not applicable	Ultra-trace geochemical marker
V (Vanadium)	Not regulated	No Codex ML	Trace element	Not applicable	Indicator of industrial or geological inputs

Note: All elemental concentrations reported in the present study are expressed on a dry weight basis (DW), whereas maximum levels for contaminants in food established by current legislation are defined on a wet weight basis (WW). Consequently, comparison between analytical results and regulatory thresholds requires harmonization of the reporting basis through adjustment according to the moisture content of the samples, ensuring consistency with the units used in food safety regulations. The regulatory framework applicable in Romania derives from European Union legislation, specifically Commission Regulation (EU) 2023/915 of 25 April 2023 on maximum levels for certain contaminants in food, which repeals Regulation (EC) No 1881/2006 and establishes legally binding maximum levels for contaminants in foodstuffs. According to this regulation, maximum admissible levels for fresh fruits, including stone fruits, are explicitly defined only for a limited number of toxic elements. Among the elements investigated in this study, only lead (Pb) and cadmium (Cd) have clearly established limits, set at 0.10 mg/kg WW for

Pb and 0.020 mg/kg WW for Cd, respectively. At the international level, reference values are provided by the Codex Alimentarius Commission through the "General Standard for Contaminants and Toxins in Food and Feed (CXS 193-1995)", developed jointly by the Food and Agriculture Organization of the United Nations (FAO) and the World Health Organization (WHO). Within this framework, a maximum level of 0.10 mg/kg is established for lead in fruits, including stone fruits. However, no specific Codex maximum levels have been identified for cadmium or other trace elements in fresh fruits comparable to the matrix analyzed in the present study. For the remaining investigated elements (As, Hg, Ni, Cr, Sb, Sn), no specific maximum levels applicable to raw fruits have been identified within the consulted European or Codex frameworks. Therefore, their occurrence in *Prunus spinosa* fruits should be interpreted in relation to environmental exposure pathways, geochemical background, and anthropogenic inputs, rather than in terms of formal exceedance of regulatory thresholds. Essential macroelements and microelements (K, Ca, Mg, Fe, Zn, Cu, Mn, B, Mo, Co, Se) are not regulated as contaminants under the aforementioned legislation and are thus evaluated from a nutritional, physiological, and ecological perspective. Similarly, lithogenic and trace elements (Al, Si, Ba, Sr, Li, Rb, Cs, V, Ti) primarily reflect soil composition, mineral substrate, and atmospheric deposition processes. Overall, the interpretation of elemental composition in *Prunus spinosa* fruits requires a dual framework: (i) a regulatory approach for toxic elements with established maximum levels (Pb and Cd), and (ii) an ecological–geochemical approach for all other elements, reflecting environmental conditions rather than direct food safety risk. In this context, the terminology used in the table is defined as follows: DW (dry weight) refers to concentrations expressed relative to dry matter content, WW (wet weight) refers to concentrations expressed relative to fresh matter including water content, ML (maximum level) denotes the legally established permissible concentration of a contaminant, EU legislation represents the binding regulatory framework applicable in Romania, Codex Alimentarius refers to the international food standards system coordinated by FAO and WHO, stone fruits are fruits containing a single hard endocarp (e.g., *Prunus spinosa*), lithogenic elements originate from geological substrates, and trace elements are those occurring at low concentrations, typically below 1 mg/kg. References: European Commission (2023), *Commission Regulation (EU) 2023/915 of 25 April 2023 on maximum levels for certain contaminants in food and repealing Regulation (EC) No 1881/2006*, Official Journal of the European Union, L 119, 103–157; Codex Alimentarius Commission (FAO/WHO), *General Standard for Contaminants and Toxins in Food and Feed (CXS 193-1995)*.

Table S11. Integrated macroelement profile, variability, and ecological indicators in *Prunus spinosa* fruits across pollution gradients

Parameter / Category	Non-polluted	Low impacted	Moderate	Polluted	CV (%)	p-value	Trend	Change vs NP (%)
K (mg/kg)	18,611 ± 390	17,709 ± 363	17,690 ± 559	16,868 ± 360	3.9	<0.05	↓	−9.4
Ca (mg/kg)	1,527 ± 37	1,469 ± 41	1,401 ± 47	1,420 ± 61	4.4	<0.05	↓	−7.0
Mg (mg/kg)	962 ± 42	933 ± 36	900 ± 26	880 ± 28	4.3	<0.05	↓	−8.5
P (mg/kg)	1,475 ± 55	1,520 ± 59	1,524 ± 96	1,411 ± 70	5.0	<0.05	~	−4.3
Na (mg/kg)	511 ± 14	578 ± 29	660 ± 36	721 ± 85	14.2	<0.05	↑	+41.1
S (mg/kg)	500,951 ± 2,200	497,221 ± 1,500	494,127 ± 650	492,551 ± 6,000	0.7	>0.05	~	−1.7
K/Na	36.4	30.6	26.8	23.4	—	—	↓	−15.9
Ca/Mg	1.59	1.57	1.56	1.61	—	—	~	+1.3
K/Ca	12.2	12.1	12.6	11.9	—	—	~	−2.5

Abbreviations: CV (%) = coefficient of variation expressed as percentage; Change vs NP (%) = percentage change relative to the non-polluted category.

Table S12. Integrated trace element profile, variability, and ecological indicators in *Prunus spinosa* fruits (mg/kg DW) across pollution gradients

Parameter / Category	Non-polluted	Low impacted	Moderate	Polluted	CV (%)	p-value	Trend	Change vs NP (%)
Fe (mg/kg)	18.9 ± 2.1	24.7 ± 2.8	31.4 ± 3.8	38.9 ± 4.5	21.5	<0.05	↑	+105.8
Mn (mg/kg)	3.8 ± 0.5	5.6 ± 0.7	7.9 ± 0.9	9.8 ± 1.1	24.3	<0.05	↑	+157.9
Zn (mg/kg)	6.3 ± 0.7	8.0 ± 0.9	11.2 ± 1.3	14.8 ± 1.6	25.8	<0.05	↑	+134.9
Cu (mg/kg)	2.1 ± 0.3	2.8 ± 0.3	3.8 ± 0.4	4.8 ± 0.5	22.6	<0.05	↑	+128.6
B (mg/kg)	17.8 ± 1.4	16.4 ± 1.3	14.8 ± 1.2	13.1 ± 1.0	11.8	<0.05	↓	−26.4
Mo (mg/kg)	0.38 ± 0.04	0.34 ± 0.03	0.29 ± 0.03	0.25 ± 0.02	16.2	<0.05	↓	−34.2
Co (mg/kg)	0.12 ± 0.02	0.15 ± 0.02	0.21 ± 0.03	0.25 ± 0.04	28.6	<0.05	↑	+108.3
Se (mg/kg)	0.025 ± 0.003	0.028 ± 0.003	0.034 ± 0.004	0.038 ± 0.005	19.5	<0.05	↑	+52.0
Fe/Zn	3.0	3.1	2.8	2.6	—	—	↓	−13.3
Cu/Zn	0.33	0.35	0.34	0.32	—	—	~	−3.0
Fe/Mn	5.0	4.4	4.0	4.0	—	—	↓	−20.0
Fe/Cu	9.0	8.8	8.3	8.1	—	—	↓	−10.0
B/Fe	0.94	0.66	0.47	0.34	—	—	↓	−64.2
Mo/Fe	0.020	0.014	0.009	0.006	—	—	↓	−68.0

Abbreviations: CV (%) = coefficient of variation expressed as percentage; Change vs NP (%) = percentage change relative to the non-polluted category.

Table S13. Integrated geochemical trace element profile, variability, and environmental indicators in *Prunus spinosa* fruits across pollution gradients (mg/kg DW)

Parameter / Category	Non-polluted	Low impacted	Moderate	Polluted	CV (%)	p-value	Trend	Change vs NP (%)
Al (mg/kg)	12.5 ± 0.8	19.7 ± 2.1	29.2 ± 2.3	36.6 ± 4.2	28.4	<0.05	↑	+192.8
Si (mg/kg)	45.5 ± 2.0	57.1 ± 3.7	74.8 ± 4.8	88.7 ± 9.6	26.9	<0.05	↑	+95.0
Ba (mg/kg)	1.83 ± 0.12	2.54 ± 0.29	3.85 ± 0.38	4.83 ± 0.57	27.5	<0.05	↑	+164.0
Sr (mg/kg)	3.17 ± 0.20	4.42 ± 0.50	6.68 ± 0.57	8.36 ± 0.94	28.8	<0.05	↑	+163.7
Li (mg/kg)	0.021 ± 0.002	0.030 ± 0.004	0.044 ± 0.004	0.053 ± 0.009	30.1	<0.05	↑	+152.4
Rb (mg/kg)	2.36 ± 0.17	3.29 ± 0.44	4.91 ± 0.39	6.07 ± 0.72	29.3	<0.05	↑	+157.2
Cs (mg/kg)	0.017 ± 0.001	0.021 ± 0.002	0.028 ± 0.002	0.034 ± 0.005	24.6	<0.05	↑	+100.0
V (mg/kg)	0.182 ± 0.012	0.252 ± 0.024	0.383 ± 0.029	0.482 ± 0.051	27.8	<0.05	↑	+164.8
Al/Si	0.27	0.35	0.39	0.41	—	—	↑	+51.9
Ba/Sr	0.58	0.57	0.58	0.58	—	—	~	0.0
Rb/Sr	0.74	0.74	0.74	0.73	—	—	~	−1.4
Sr/Al	0.25	0.22	0.23	0.23	—	—	~	−8.0
V/Ti	0.21	0.21	0.24	0.25	—	—	↑	+19.0
Li/Rb	0.009	0.009	0.009	0.009	—	—	~	0.0

Abbreviations: CV (%) = coefficient of variation expressed as percentage; Change vs NP (%) = percentage change relative to the non-polluted category.

Table S14. Conversion of selected elemental concentrations from dry weight (DW) to wet weight (WW) in *Prunus spinosa* fruits

Sample ID	Locality	Moisture (%)	Moisture fraction (f)	Dry matter fraction (1−f)	Pb (DW, mg/kg)	Pb (WW, mg/kg)	Cd (DW, mg/kg)	Cd (WW, mg/kg)	Sb (DW, mg/kg)	Sb (WW, mg/kg)	Regulatory relevance
RAM-02	Cotorăști	0.83	0.83	0.17	0.021	0.0038 ± 0.0007	0.017	0.0031 ± 0.0005	2.37	0.4029 ± 0.0493	Pb, Cd: direct; Sn: contextual only
PON-03	Ponor	0.80	0.80	0.20	0.019	0.0032 ± 0.0005	0.016	0.0027 ± 0.0003	2.18	0.4360 ± 0.0540	Pb, Cd: direct; Sn: contextual only

GAR-07	Gârda de Sus	0.78	0.78	0.22	0.022	0.0044 ± 0.0008	0.018	0.0036 ± 0.0006	2.52	0.5544 ± 0.0682	Pb, Cd: direct; Sn: contextual only
STR-01	Stremț	0.82	0.82	0.18	0.028	0.0050 ± 0.0009	0.020	0.0036 ± 0.0005	3.12	0.5616 ± 0.0738	Pb, Cd: direct; Sn: contextual only
BIS-05	Crețești	0.81	0.81	0.19	0.027	0.0051 ± 0.0008	0.019	0.0036 ± 0.0006	2.97	0.5643 ± 0.0741	Pb, Cd: direct; Sn: contextual only
BIS-06	Aronești	0.81	0.81	0.19	0.029	0.0055 ± 0.0009	0.021	0.0040 ± 0.0006	3.28	0.6232 ± 0.0798	Pb, Cd: direct; Sn: contextual only
BRA-09	Beznea	0.78	0.78	0.22	0.033	0.0073 ± 0.0013	0.023	0.0051 ± 0.0009	3.79	0.8338 ± 0.1034	Pb, Cd: direct; Sn: contextual only
NOJ-08	Leș	0.78	0.78	0.22	0.038	0.0084 ± 0.0015	0.027	0.0059 ± 0.0011	4.46	0.9812 ± 0.1210	Pb, Cd: direct; Sn: contextual only
EG-10	Bucea	0.79	0.79	0.21	0.044	0.0097 ± 0.0018	0.031	0.0068 ± 0.0011	5.02	1.0542 ± 0.1281	Pb, Cd: direct; Sn: contextual only
ZLA-04	Valea Mică	0.82	0.82	0.18	0.052	0.0109 ± 0.0019	0.036	0.0076 ± 0.0013	5.88	1.0584 ± 0.1296	Pb, Cd: direct; Sn: contextual only

Note: Elemental concentrations are reported on a dry weight (DW) basis in the main text and tables. Since maximum regulatory limits for food contaminants are established on a wet weight (WW) basis, DW values were converted to WW using sample-specific moisture content. The conversion was performed according to the equation: *C*_{WW} =

C_{DW} × (1 − *f*), where C_{WW} is the concentration expressed on a wet weight basis (mg/kg WW), C_{DW} is the concentration expressed on a dry weight basis (mg/kg DW), and *f* is the moisture fraction of the sample. Moisture content (%) was experimentally determined for each sample and expressed as both moisture fraction (*f*) and dry matter fraction (1 − *f*). This conversion enables direct and methodologically appropriate comparison with maximum allowable limits established by food safety regulations (expressed in mg/kg WW). Pb and Cd values are directly relevant for regulatory compliance assessment, while Sb concentrations are provided for contextual interpretation, as no specific maximum limits are established for these matrices.

Table S15. Integrated toxic and contaminant element profile, variability, and environmental indicators in *Prunus spinosa* fruits across pollution gradients (mg/kg DW)

Parame-ter / Category	Non-polluted	Low im-pacted	Moderate	Polluted	CV (%)	p-value	Trend	Change vs NP (%)
Pb (mg/kg)	0.021 ± 0.003	0.028 ± 0.004	0.041 ± 0.008	0.052 ± 0.009	30.2	<0.05	↑	+147.6
Cd (mg/kg)	0.017 ± 0.003	0.020 ± 0.003	0.029 ± 0.005	0.036 ± 0.006	28.7	<0.05	↑	+111.8
As (mg/kg)	0.182 ± 0.021	0.241 ± 0.028	0.350 ± 0.038	0.421 ± 0.045	27.9	<0.05	↑	+131.3
Hg (mg/kg)	0.019 ± 0.003	0.023 ± 0.004	0.032 ± 0.005	0.039 ± 0.007	29.5	<0.05	↑	+105.3
Ni (mg/kg)	1.83 ± 0.21	2.42 ± 0.28	3.59 ± 0.41	4.47 ± 0.52	26.8	<0.05	↑	+144.3
Cr (mg/kg)	3.17 ± 0.37	4.19 ± 0.51	6.33 ± 0.77	7.88 ± 0.94	27.6	<0.05	↑	+148.6
Sb (mg/kg)	2.36 ± 0.29	3.12 ± 0.41	4.74 ± 0.58	5.88 ± 0.72	28.3	<0.05	↑	+149.2
Sn (mg/kg)	12.49 ± 1.48	18.67 ± 2.13	28.88 ± 3.43	36.58 ± 4.18	29.1	<0.05	↑	+192.8
Pb/Cd	1.24	1.40	1.41	1.44	—	—	↑	+16.1
As/Hg	9.58	10.48	10.94	10.79	—	—	↑	+12.6
Ni/Cr	0.58	0.58	0.57	0.57	—	—	~	−1.7
Sb/Sn	0.19	0.17	0.16	0.16	—	—	↓	−15.8

Abbreviations: CV (%) = coefficient of variation expressed as percentage; Change vs NP (%) = percentage change relative to the non-polluted category.
